# Risk of Exposure to COVID-19: Visit Duration Data Can Inform Our Daily Activities Choices: An Epidemiological Investigation Using Community Mobility Data from the Metropolitan Area of Genoa, Italy

**DOI:** 10.3390/ijerph18094632

**Published:** 2021-04-27

**Authors:** Cristina Oliva, Giampiero Favato

**Affiliations:** Institute of Leadership and Management in Health, Kingston Business School, Kingston University, Kingston-upon-Thames KT2 7LB, UK; c.oliva@kingston.ac.uk

**Keywords:** COVID-19, risk, exposure, visit, duration

## Abstract

COVID-19 spreads mainly among people who are in close contact. Policymakers mostly resorted to normative measures to limit close contacts and impose social distancing. Our study aimed to estimate the risk of exposure to COVID-19 by location and activity in crowded metropolitan areas. The risk of exposure to COVID-19 was defined as the product of crowding (people within a six feet distance) and exposure duration (fraction of 15 min). Our epidemiological investigation used aggregated and anonymized mobility data from Google Maps to estimate the visit duration. We collected visit duration data for 561 premises in the metropolitan area of Genoa, Italy from October 2020 to January 2021. The sample was then clustered into 14 everyday activities, from grocery shopping to the post office. Crowding data by activity were obtained from pre-existing building norms and new government measures to contain the pandemic. The study found significant variance in the risk of exposure to COVID-19 among activities and, for the same activity, among locations. The empirical determination of the risk of exposure to COVID-19 can inform national and local public health policies to contain the pandemic’s diffusion. Its simple numerical form can help policymakers effectively communicate difficult decisions affecting our daily lives. Most importantly, risk data by location can help us rethink our daily routine and make informed, responsible choices when we decide to go out.

## 1. Introduction

Policymakers mainly resorted to normative measures to mitigate the individual risk of exposure to COVID-19. Over the last 12 months, the Italian Government promulgated 73 Acts containing urgent measures to contain the pandemic [1]. These norms imposed on individuals the trade-off between mitigation of the risk of exposure and personal freedom. Stay home (lockdown), avoid crowds (limited opening hours and restricted access to stores), and wear a mask became new legally binding constraints to our everyday life. A constant, systematic media campaign made everyone aware of what not to do, and the consequences of breaking the law. For the first time, the Prime Minister asked social media influencers to promote the adherence to public health policies, leveraging the connection to the civic sense of younger users [2].

When people are allowed to go out, what can they do to reduce their risk of exposure to COVID-19? Which activity is riskier? Within the same activity, are there premises where the risk of exposure is lower than others?

The aim of our study was to estimate the risk of exposure to COVID-19 by location and activity in crowded metropolitan areas.

Although social and leisure activities have been identified as significant public health hazards related to the diffusion of COVID-19, the Centers for Disease Control (CDC) admittedly “cannot provide the specific risk level for every activity in every community”. No method or dataset in the extant literature can help individuals make informed decisions about the risk of exposure to COVID-19 when they decide to go out.

Our epidemiological investigation used for the first time the newly available mobility data to estimate the risk of exposure to COVID-19 in crowded retail premises of Genoa’s metropolitan area (Italy). The newly obtained granularity of risk data could inform people’s daily choices when deciding to go out, increasing the individual acceptance of containment measures and reducing the exposure to COVID-19 at a personal and community level.

## 2. Materials and Methods

### 2.1. Risk of Exposure to COVID-19: A Working Definition

COVID-19 spreads mainly among people who are in close contact [3]. Factors to consider when defining close contact include proximity (closer distance likely increases exposure risk) and exposure duration (longer exposure time likely increases exposure risk). Although data are still limited, 15 cumulative minutes of exposure at a distance of 6 feet or less can be used as an operational definition for close contact [4]. As recommended by CDC, the determination of close contact should generally be made irrespective of whether the contact was wearing respiratory personal protective equipment (PPE). The impact of wearing a mask on reducing the exposure risk for specific daily activities is addressed in the “Discussion” Section 4. From the CDC’s definition of closed contact, we derived a working definition of risk of exposure to COVID-19 for daily activities:

Risk of exposure = crowding × visit duration(1)

### 2.2. The Measurement of Crowding

To determine the exact number of people standing in the 10.4 square meters (approximately to the area of a circle of 6 feet radius) around you at any given time is virtually impossible. What is possible is to estimate the maximum number of people you should expect around you in any public office or retail premise. In Italy, maximum crowding standards are regulated by the UNI10339 norm, which sets the maximum number of people allowable for design purposes, for each square meter of floor area, concerning various categories of public offices and retail premises [5]. To guarantee the resumption of activities after the first lockdown phase, the maximum crowding standard attributed to commercial establishments was set at 13.3 m^2^ per person (example: three people can enter a 40 m^2^ room) [6] In May 2020, the National Institute for Occupational Accident Insurance (INAIL) produced a technical document about coffee shops and restaurants. It set the maximum crowding standard at 4 square meters per person [7]. We used both sets of crowding norms as multiplicands to determine the risk of exposure to COVID-19 before and after the Government’s containment measures.

### 2.3. New Data: The Measure of Visit Duration

The real methodological issue was estimating the time multiplier: how long do people stay in a specific store or premise in a given community? A new feature of Google Maps allowed collecting data on the mean visit duration by individual retail premise. Google made visit duration data available in October 2020, only for store with an acceptable level of customers’ daily traffic. Due to the Covid-19 limitations to mobility, we waited approximately three months (30 December 2020) before collecting visit duration data from a significant number of retail stores by type of activity.

This new feature shows how much time customers typically spend in a specific store. Visit duration is based on customer visits patterns over the past several weeks and is expressed in units of time (minutes). Most retail stores show the visit duration as a range (e.g., 90–180 min), while food supermarkets indicate a mean value (e.g., 20 min) [8].

Data were collected from all the retail activities resident in the Genoa metropolitan area which were visible in Google Map and reported visit duration time. Google does not report visit duration for those activities which do not generate a reliable number of daily mobility data. We manually collected visit duration data for 561 retail activities, banks and public offices located by Google Maps in the metropolitan area of Genoa, Italy. The sample was then clustered into 14 everyday activities, from grocery shopping to going to the post office. 

Interpreting mobility data in metropolitan areas requires an in-depth understanding of the urbanism and road mapping of the selected area. The choice of the location was determined by the fact that one of the Authors was born and raised in the metropolitan area of Genoa. Data collected for the study, including individual location data and a data dictionary defining each field in the set are available in the online Supplementary Material.

### 2.4. Statistical Analysis

We calculated the median visit duration by activity for both the upper and the lower limit of the range using the statistical software MedCalc (MedCalc Software Ltd, Ostend, Belgium). We used these values as multipliers to estimate the risk of exposure to COVID-19 by type of activity. The choice of median values is consistent with Google’s method to calculate mobility data changes across different categories of places [9]. The descriptive statistics are reported in the online Supplementary Material. 

The contact risk of Covid-19 transmission was defined by CDC as a deterministic model, the product of one constant value (crowding) by one variable (duration). To estimate the risk of exposure by activity, we used the median visit duration by location type as reported by Google Map. Data on visit duration by store were non-random, since we did not use a sample, and non-normally distributed. We tested the significance of the estimated parameter (median visit duration by retail activity, ϕ_i_) by testing the hypothesis [10]:

Hypothesis 0 (H0): ϕ_i_ = 0.

Hypothesis 1 (H1): ϕ_i_ ≠ 0.

(ϕ_i_ − 0)/(Std.error ϕ_i_) ≅ t_n − m_ (2)

where m is the number of parameters.

For α = 0.05

(ϕ_i_ − 0)/(Std.error ϕ_i_) > 2 (3)

To estimate the standard error of the median, visit duration data by retail activity were resampled with replacement 1000 times using the statistical software Resampling Stat in Excel version 2 (Resampling Stats, Inc., Arlington, VA, USA). The significance of the derived parameter, the median value of visit duration, is reported in Table 1: all parameters resulted significantly different from 0 (α = 0.05).

**Figure 1:**
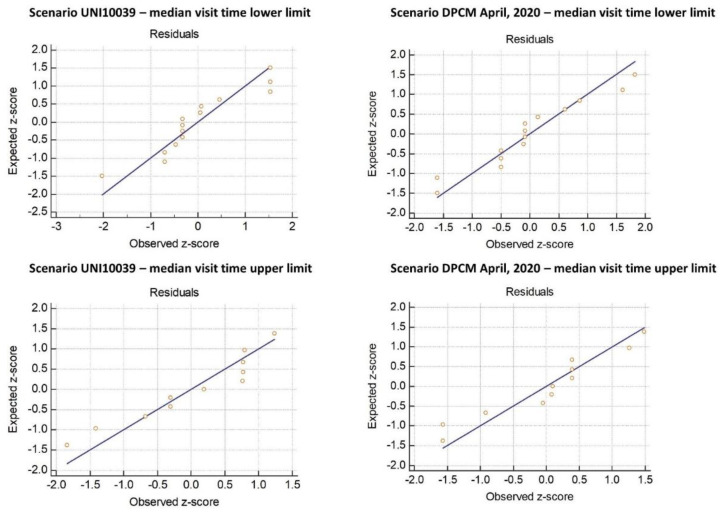
Regression of median visit duration against predicted contact risk for the four scenarios: controlling for normality of residuals.

We then tested the accuracy of the contact risk model by regressing the median visit duration by store type against the predicted risk values and checking for normality of residuals. The normal plot of residuals for the four scenarios (crowding norm UNI10339 and DPCM 2020, lower and upper median values of visit duration by retail activity) are reported in Figure 1. In all four scenario the hypothesis of normal distribution of residuals could be accepted (Kolmogorov-Smirnov test).

The estimated predictors (median visit time by retail activity) was regressed against the predicted contact risk for four scenarios: crowding norm UNI10039 and DPCM 2020, lower and upper values of visit duration. The figure shows the Q-Q plot distribution of residuals. For all scenarios the hypothesis of normal distribution of residual could be accepted (Kolmogorov-Smirnov test).

## 3. Results

### 3.1. Study Sample and Inputs to the Model

Food supermarkets (n = 170), post offices (n = 57) banks (n = 38), and pharmacies (n = 35) were among the most represented locations in the dataset (53.5% of total). This is not surprising, since they fulfil vital needs of our daily life and they have not been subject to forced closures even during the first and second lockdown (in April and December 2020, respectively). Social activities, such as pizza restaurants (n = 41), fine dining (n = 39), pubs (n = 22), fast-food (n = 19), and coffee shops (n = 14), represented 24% of the total locations included in the sample, a true testament of the importance of personal contact in our culture. More controversial activities, such as hairdressers (n = 14) and gyms (n = 10) were also significantly represented in the sample. 

The median visit duration was reported as a range (upper and lower limits) for 11 out of 14 retail activities: for grocery shops, pharmacies and gas stations, Google Map displayed only the median average visit duration. The median visit time’s confidence intervals offer a plausible explanation to this reporting difference. While the dispersion is narrow for in-and-out daily activities (such as grocery shopping or filling-up the car at a gas station), the variance of time spent in other activities can be better expressed as a range. For example, a quick lunch in a restaurant takes on average less time than a three-course dinner. Table 2 reports detailed information on the study sample and the inputs used to calculate the risk of exposure to COVID-19 by retail activity. 

A simple vertical and horizontal analysis of visit duration data by activity provides valuable insights on the potential risk of exposure to COVID-19 when we go out.

The vertical analysis shows that we spend at least one hour in restaurants and pubs, a visit duration which is three to six-fold higher than any other activity. Moreover, the median visit duration to restaurants and gyms more than doubles at the upper limit of the range, providing a clear indication that social activities and indoor exercise should be, and are, a key priority for the containment of the diffusion of COVID-19.

The horizontal comparison between lower and upper limits of the median visit time range reflects our collective behavior’s typical traits, making the differences more credible. For example, a quick espresso at the bar counter takes about 17 min, while an aperitif followed by an animated discussion about football can go on for an hour. Even fast food can be not so fast in Italy: a hamburger gobbled up between two lectures takes about 25 min, but if we sit down to plan the evening with our friends, then the median duration of the visit can almost double.

We used both publicly available norms (UNI10339 and DPCM, 19 April 2020) to define the maximum crowding standard (number of people per square meter) expected for each activity included in our sample.

### 3.2. Absolute and Relative Risk of Exposure to COVID-19 by Retail Activity under Crowding Norm UNI10339 (Antecedent the First Lockdown in March 2020)

The absolute values show a quite alarming variance of risk exposure to COVID-19 depending on our choice of activity and time spent on a retail premise. The range of exposure goes from a minimum of 1.39 when we stop at a gas station to a record high of 68.64 if we decide to reward ourselves with a nice dinner out in a fine dining restaurant. Within the same activity, the risk of exposure for a quick work-out in a gym is 9.1, but it can more than double for prolonged fitness training (20.8). It is even worse for coffee shops: an espresso at the counter gives an exposure of 9.7, while our beloved habit of continuing an animated conversation at a table can cost us a risk over three times higher (33.3). 

Daily errands such as grocery shopping or going to the bank, pharmacy or post office seem to carry a much lower risk of exposure to COVID-19 (ranging from just above 2 to 4). This is a relief, not only because such activities are indispensable to our daily lives, but also because they are an essential part of older people’s daily routine, most vulnerable to COVID-19 infection [11]. 

Table 3 summarizes the absolute and relative risk of exposure to COVID-19 by retail activity before the first lockdown initiated on 9 March 2020.

### 3.3. Absolute and Relative Risk of Exposure after the First Lockdown (Crowding Norm DPCM, 19 April 2020)

**Figure 2:**
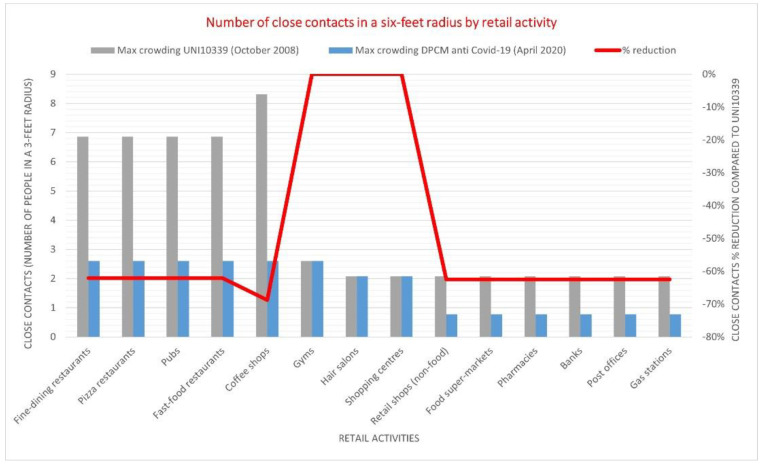
Number of close contacts in a 6-feet radius by retail activity.

Following the first lockdown, the Italian Government decided to reduce the crowding standards for all premises open to the public. Figure 2 shows the reduction in the number of close contacts expected in a three-foot radius compared to the previous norm (UNI10339). The drop exceeded 60% for most daily activities, while only gyms, hair salons, and shopping centers were unaffected by the new norm.

Left scale: the grey columns report the maximum number of people allowed by the UNI10339 (October 2008) norm in a six-feet radius space by retail activity. The blue columns report the maximum number of people allowed by the Prime Ministerial Decree (DPCM) anti-COVID (April 2020) by retail activity. Right scale: the red line shows the percent reduction in the number of closed contacts determined by the DPCM by comparison with the UNI10339 norm.

Table 4 summarizes the impact of the reduction of crowding standards on the risk of exposure to COVID-19 after the DPCM in April 2020. In summary, the new crowding norm introduced after lockdown substantially confirmed a three-tier risk structure for daily activities:

(1) HIGH RISK (minimum relative risk >10): fine-dining restaurants, pizza restaurants, pubs and gyms;
(2) MEDIUM RISK (minimum relative risk >5, but likely to exceed the threshold of HIGH RISK based on the duration of the visit): fast-food restaurants, coffee shops, hair salons, shopping centers;
(3) LOW RISK (relative risk always <5): retail shops (non-food), grocery supermarkets, pharmacies, banks, post office and gas stations.

## 4. Discussion

This study used the mean visit duration for the first time, a new feature of Google Maps to determine the risk of exposure to COVID-19 for many daily activities in a specific community, the metropolitan area of Genoa, Italy. The study found a significant variance in the risk of exposure among different activities and, for the same activity, among different locations. Since the study was informed by publicly available mobility and crowding data, this simple method could inform individual choices when deciding to go out, containing the risk of COVID infection by merely avoiding or reducing exposure to crowded locations. Since this study is the first of a kind, we should answer some fundamental methodological questions before recommending its wider adoption. The first question concerns the appropriateness of mobility data to inform COVID-19 analysis of risk exposure. Google publicly discloses aggregated, anonymised GPS location data at metropolitan level containing users’ density and proximity data. Accepted applications of location data include changes to population-level mobility and clustered behaviours useful to understand the risk of close contact, retrace likely diseases introduction and, most importantly, to inform the projections of risk of disease [12]. The second question is about the use of crowding standards, which measure the maximum number of people allowable in a premise rather than the actual number of individuals in the store at any given time. Actual crowding data can be obtained by learning location profiles from heterogeneous mobility datasets based on gravity models [13]. Collecting individual mobility data requires massive computational capacity and a standard for exchanging data between mobile operators and regulators (Mobility Data Specification). The outcomes of gravity models can inform public health policies but are of little help when making individual decisions about going out. Conversely, crowding standards are easier to understand for the general public: based on the DPCM norm, you should expect at least one but no more than three people in your closed contact risk area, a circle of six feet radius. If you can see more than three people around you, you know that the premise is overcrowded. The third question concerns the accuracy of predicting the risk of exposure to COVID-19 by activity based on crowding standards and the visit duration. 

Predicting the risk of exposure to COVID-19 by activity based on crowding standards and the visit duration accurately reflects the containment priorities and emergency measures in place so far by the Italian Government. Most of the activities have been affected by a drastic reduction of crowding standards, after the DPCM in April 2020. Restaurants and pubs have been closed down during the lockdown in April and December. Their opening hours have been drastically reduced across the period, with no service in the premises allowed after 6 p.m. Gyms are still closed. Coffee shops, fast-food restaurants, and hair salons have also been closed down during lockdown, and their opening hours reduced as well when re-opening has been allowed. Retail shops (non-food) were closed during the lockdown, but their activity resumed as usual when the lockdown was lifted. Activities showing the lowest risk level, such as grocery supermarkets, pharmacies, banks, post offices and gas stations, have never been closed and their store hours never reduced. 

When we include the use of facial masks, the assessment of exposure to COVID-19 based on crowding standards and visit duration may have underestimated the risk for social activities, already ranked at the highest level of concern. When eating a meal or sipping a coffee, you necessarily put your mask down. Considering that face masks may significantly reduce the exposure to the virus [14], the risk of exposure to COVID-19 for restaurants of any kind (including fast food), pubs, and coffee shops can be greater than expected. Also, the notion of crowding standards may have contributed to understate the risk of social activities. Crowding standards account for the maximum allowable people per square meter, but they do not tell us how long the same person stays at least 15 min in a six-foot radius. Social activities, such as dining out, sitting at a coffee shop, or having a burger meal at the table carry a higher likelihood to have the same individuals around for longer than fifteen minutes than moving along the aisle of a supermarket or making an enquiry at the desk of a bank or a post office. Exercising at a gym or having your hair done at the hairdresser are also likely to carry a higher risk than filling a prescription at the pharmacy or refuelling your car. We can conclude that the use of face masks and the likelihood of permanence in a six feet radius does not change the distribution of the risk of exposure to COVID-19 as found by our study.

The risk of exposure to COVID-19, measured as the product of crowding standards times median visit duration, can be useful to inform public health policies and individual decision about going out.

The intuitive, numeric form that we chose to define the risk of exposure can help policymakers effectively communicate the urgency of drastic containment measures to limit the diffusion of COVID-19. These measures are currently imposed on the general population as mandatory norms, without a transparent explanation of why these prohibitions are necessary preventive measures and not just arbitrary limitations of individual freedom. Prohibitions are generally poorly tolerated and, in the long run, the adherence to the new norms on daily lifestyle sharply decreases [15]. As an example, eating out is an essential part of the Italian lifestyle. The prolonged closure of restaurants, followed by a severe limitation of their opening hours (take-away and delivery only after 6 p.m.) has generated a vast dissatisfaction in the population, craving for social contact after a full year of distancing. It is conceivable that individuals would react differently if they were told that dining out carries the highest absolute risk of exposure to COVID-19 (from 10 to 26), fifty times higher than refuelling the car at a gas station or 20 times higher than grocery shopping. The use of a numerical indicator would have probably placated sooner the controversy about the re-opening of gyms and hair salons, which may carry a risk of exposure similar to dining out in case of prolonged duration of the visit [16,17]. 

This study’s main contribution is defining a single number to indicate the relative risk of exposure to COVID-19 for most of the activities that we need to perform in our daily lives. Moreover, for the same activity, it allows us to choose between different locations in our community where the absolute risk of exposure is lower. For example, when you decide to go to the post office, Google Maps can help you choose the location with the shortest mean duration of the visit. In Genoa’s metropolitan area, the post office in Via Dante shows a mean visit duration of 45 min, while the post office in Via Ilva has a mean visit duration of just 15 min. The two offices are both downtown, only 700 m away from each other: a ten-minute walk can bring the risk of exposure to COVID-19 down to one third [18].

This research presents some limitations. The study is subject to a risk of selection bias in the population for whom data is available, limited to smartphone users who have turned on the Location History setting, which is off by default. This is a general limitation imposed by the use of GPS mobility data [19]. Spatially and temporally aggregated mobility data also do not capture differences in how individuals use their phones, making unfeasible any further cohort analysis (e.g., by users’ age, gender or income). No data privacy issue is associated with the mobility data used to inform our risk model. Google Map publicly provides the duration of visit data by premise in a strictly aggregated and anonymised form. No personally identifiable information, such as an individual’s location, contacts or movement, was made available at any point.

## 5. Conclusions

Our study enables everyone to understand the potential risk of going out and to make a responsible choice of daily activities in the community of residence.

Firstly, we used a working definition of risk of exposure leading to a simple, numerical value. Everybody understands the absolute and relative difference between two numbers: as an example, ten is simply five times higher than two.

Secondly, the definition of the two main factors of risk, crowding and visit duration, is intuitive.

Crowding refers to the number of people standing in a circle of three feet radius centred around you. The visit duration simply refers to the number of minutes you spend on average in a store or public office. The new feature of Google Maps allows everyone to be informed about the mean visit duration for many locations in their community. Since the crowding standards are the same for each type of activity, this simple, easy to get information can guide everyone’s daily routine activities.

The possibility to measure the risk of exposure by a single location can inform national and public policies aimed to contain the COVID-19 pandemic. More importantly, using a local, numeric value to define the risk can help policymakers make explicit the rationale of measures that have a hard impact on the population’s social life, improving adherence over time.

The most significant impact of this research is to make aware individuals of the absolute and relative risk of exposure to COVID-19, empowering them to make active choices when they decide to go out.

The study’s findings suggest that the new data on the visit duration provided by Google Map can help understand the risk of exposure to COVID-19 associated with the most common activities in our daily life. The empirical determination of risk defined in our study can inform national and local public health policies to contain the pandemic’s diffusion. Its simple numerical form can help policymakers effectively communicate difficult decisions concerning our daily lives, justifying their rationale using a language that everyone can understand. Lastly, risk data by location can help us rethinking our daily routine and making informed, responsible choices when we decide to go out. 

## Figures and Tables

**Table 1:** Significance of estimated parameter: median visit duration by retail activities.

Retail Activities	Median Visit Duration by Retail Activity in the Metropolitan Area of Genoa (Italy) Source: Google Maps 30 December 2020									
	Lower Limit of the Range					Upper Limit of the Range				
	Sample (n)	Median Visit Duration (Minutes)	Variance	Standard Error of the Median (Resampled)	Significance of Median Values (α = 0.05)	Sample (n)	Median Visit Duration (Minutes)	Variance	Standard Error of the Median (Resampled)	Significance of Median Values (α = 0.05)
Pubs	22	60	3.8	3.8	15.6 **	22	120	167.2	12.9	9.3 **
Pizza restaurants	41	60	0.8	0.8	73.1 **	41	120	154.4	12.4	9.7 **
Fine-dining restaurants	36	60	12.6	12.6	4.8 **	39	150	222.4	14.9	10.1 **
Gyms	10	53	13.9	13.9	3.8 **	10	120	143.7	12.0	10.0 **
Hair salons	14	30	6.2	6.2	4.8 **	14	90	305.3	17.5	5.2 **
Fast-food restaurants	19	25	4.2	4.2	5.9 **	11	45	64.6	8.0	5.6 **
Food supermarkets *	170	20	1.8	1,8	11.1 **	N/A	N/A	N/A	N/A	N/A
Shopping centres	16	20	2.98	1.73	11.6 **	16	60	93.5	9.7	6.2 **
Retail shops (non-food)	13	20	2.4	1.5	13.0 **	86	25	0.4	0.7	38.2 **
Coffee shops	14	18	10.6	3.3	5.4 **	10	60	38.7	6.2	9.6 **
Banks	38	15	0.1	0.4	38.9 **	14	45	0.1	0.3	134.3 **
Pharmacies *	35	15	6.09	2.5	6.1 **	N/A	N/A	N/A	N/A	N/A
Post offices	57	15	6.2	2.49	6.0 **	11	45	0.1	0.2	189.8 **
Gas stations *	20	10	2.4	1.5	13.6 **	N/A	N/A	N/A	N/A	N/A

* Google Maps only reported the average visit duration. ** Significantly different from 0 (values >2 at α = 0.05).

**Table 2:** Study sample and inputs to the model.

Retail Activities	Visit Duration by Retail Activity in the Metropolitan Area of Genoa, Italy (Source: Google Maps, 30 December 2020)												Crowding Standard (Maximum Number of People Allowed Per Square Meter)	
	Lower Limit of the Range						Upper Limit of the Range							
	Sample (n)	Median Visit Duration (Minutes)	95% Confidence Interval of the Median		MIN Median Visit Duration (Minutes)	MAX Median Visit Duration (Minutes)	Sample (n)	Median Visit Duration (Minutes)	95% Confidence Interval of the Median		MIN Median Visit Duration (Minutes)	MAX Median Visit Duration (Minutes)	UNI10339 (October 2008)	DPCM Anti Covid-19 (April 2020)
Pubs	22	60	45	60	15	90	22	120	120	150	90	180	0.66	0.250
Pizza restaurants	41	60	60	60	20	120	41	120	120	150	90	180	0.66	0.250
Fine-dining restaurants	36	60	60	90	30	90	39	150	120	150	60	180	0.66	0.250
Gyms	10	53	20	60	5	90	10	120	90	120	45	150	0.66	0.250
Hair salons	14	30	25	45	10	60	14	90	60	123	60	180	0.80	0.250
Fast-food restaurants	19	25	15	30	10	45	11	45	45	65	30	90	0.25	0.250
Food supermarkets *	170	20	20	20	5	45	N/A	N/A	N/A	N/A	N/A	N/A	0.20	0.200
Shopping centres	16	20	20	27	10	30	16	60	60	90	45	90	0.20	0.200
Retail shops (non-food)	13	20	18	25	15	30	86	25	25	25	10	90	0.20	0.075
Coffee shops	14	18	15	25	10	30	10	60	45	60	45	90	0.20	0.075
Banks	38	15	15	15	10	30	14	45	45	45	45	90	0.20	0.075
Pharmacies *	35	15	15	15	10	20	N/A	N/A	N/A	N/A	N/A	N/A	0.20	0.075
Post offices	57	15	15	20	10	25	11	45	45	45	45	60	0.20	0.075
Gas stations *	20	10	10	10	10	15	N/A	N/A	N/A	N/A	N/A	N/A	0.20	0.075

* Google Maps only reported the average visit duration.

**Table 3:** Absolute and relative risk of exposure to COVID-19 before the first lockdown (crowding norm UNI10339, October 2008).

Retail Activities	Median Visit Duration (Minutes)		Median Visit Duration as a Fraction of 15 min		Crowding (People in the Contact Area)			Absolute Risk of Exposure to COVID-19		Relative Risk of Exposure to COVID-19 (Gas Stations = 1)	
	Lower Limit	Upper Limit	Lower Limit	Upper Limit	UNI10339 Crowding Standard (People Per Square Meter)	Close Contact Area in Square Meters (CDC, Oct 2020)	Max of People in the Contact Area	Lower Limit	Upper Limit	Lower Limit	Upper Limit
	a	b	a/15	b/15	c	d	c×d	(a/15)×c×d	(b/15)×c×d	[(a/15)×c×d]/1.39	[(b/15)×c×d]/1.39
/Fine-dining restaurants	60	150	4.00	10.00	0.7	10.4	6.86	27.46	68.64	19.8	49.4
Pizza restaurants	60	120	4.00	8.00	0.7	10.4	6.86	27.46	54.91	19.8	39.5
Pubs	60	120	4.00	8.00	0.7	10.4	6.86	27.46	54.91	19.8	39.5
Fast-food restaurants	25	45	1.67	3.00	0.7	10.4	6.86	11.44	20.59	8.2	14.8
Coffee shops	18	60	1.17	4.00	0.8	10.4	8.32	9.71	33.28	7.0	23.9
Gyms	53	120	3.50	8.00	0.3	10.4	2.60	9.10	20.80	6.5	15.0
Hair salons	30	90	2.00	6.00	0.2	10.4	2.08	4.16	12.48	3.0	9.0
Shopping centres	20	60	1.33	4.00	0.2	10.4	2.08	2.77	8.32	2.0	6.0
Retail shops (non-food)	20	25	1.33	1.67	0.2	10.4	2.08	2.77	3.47	2.0	2.5
Food supermarkets *	20		1.33		0.2	10.4	2.08	2.77		2.0	
Pharmacies *	15		1.00		0.2	10.4	2.08	2.08		1.5	
Banks	15	45	1.00	3.00	0.2	10.4	2.08	2.08	6.24	1.5	4.5
Post offices	15	45	1.00	3.00	0.2	10.4	2.08	2.08	6.24	1.5	4.5
Gas stations *	10		0.67		0.2	10.4	2.08	1.39		1.0	

* Google Maps only reported the average visit duration.

**Table 4:** Absolute and relative risk of exposure after the first lockdown (crowding norm DPCM, April 2020).

Retail Activities	Median Visit Duration (Minutes)		Median Visit Duration as a Fraction of 15 min		Crowding (People in the Contact Area)			Absolute Risk of Exposure to COVID-19		Relative Risk of Exposure to COVID-19 (Gas Stations = 1)	
	Lower Limit	Upper Limit	Lower Limit	Upper Limit	DPCM Anti Covid-19, Crowding Standard (People Per Square Meter)	Close Contact Area in Square Meters (CDC, October 2020)	Max Number of People in the Contact Area	Lower Limit	Upper Limit	Lower Limit	Upper Limit
	a	b	a/15	b/15	c	d	c×d	(a/15)×c×d	(b/15)×c×d	[(a/15)×c×d]/0.52	[(b/15)×c×d]/0.52
Fine-dining restaurants	60	150	4.00	10.00	0.250	10.40	2.60	10.40	26.00	20.0	50.0
Pizza restaurants	60	120	4.00	8.00	0.250	10.40	2.60	10.40	20.80	20.0	40.0
Pubs	60	120	4.00	8.00	0.250	10.40	2.60	10.40	20.80	20.0	40.0
Fast-food restaurants	25	45	1.67	3.00	0.250	10.40	2.60	4.33	7.80	8.3	15.0
Coffee shops	18	60	1.17	4.00	0.250	10.40	2.60	3.03	10.40	5.8	20.0
Gyms	53	120	3.50	8.00	0.250	10.40	2.60	9.10	20.80	17.5	40.0
Hair salons	30	90	2.00	6.00	0.200	10.40	2.08	4.16	12.48	8.0	24.0
Shopping centres	20	60	1.33	4.00	0.200	10.40	2.08	2.77	8.32	5.3	16.0
Retail shops (non-food)	20	25	1.33	1.67	0.075	10.40	0.78	1.04	1.30	2.0	2.5
Food supermarkets *	20		1.33		0.075	10.40	0.78	1.04		2.0	
Pharmacies *	15		1.00		0.075	10.40	0.78	0.78		1.5	
Banks	15	45	1.00	3.00	0.075	10.40	0.78	0.78	2.34	1.5	4.5
Post offices	15	45	1.00	3.00	0.075	10.40	0.78	0.78	2.34	1.5	4.5
Gas stations *	10		0.67		0.075	10.40	0.78	0.52		1.0	

* Google Maps only reported the average visit duration.

## Data Availability

All data supporting this study can be found in the Supplementary Materials.

## Supplementary Materials

The following are available online at https://www.mdpi.com/article/10.3390/ijerph18094632/s1. All data supporting this study.
