# Supplementary material for: Risk of Exposure to COVID-19: Visit Duration Data Can Inform Our Daily Activities Choices: An Epidemiological Investigation Using Community Mobility Data from the Metropolitan Area of Genoa, Italy
Source: Int J Environ Res Public Health. 2021 Apr 27;18(9):4632. doi: 10.3390/ijerph18094632 (PMC8123828; doi:10.3390/ijerph18094632)

Supplementary Material

Table of Contents

Definition of close contact risk of Covid-19 4

Maximum crowding standard by retail activity 5

UNI 10339 norm. 5

DPCM anti Covid-19 5

Mean visit duration in retail shopping premises: the Google Maps data 7

Visit duration 7

Food supermarkets (n=170) 8

Shopping centres (n=16) 12

Coffee shops (n=14) 12

Fast-food restaurants (n=19) 13

Pubs (n=22) 14

Pizza restaurants (n=41) 15

Fine dining restaurants (n=39) 16

Retail shops non food (n=86) 17

Gyms (n=10) 19

Hair saloons (n=14) 19

Post offices (n=57) 20

Banks (n=38) 22

Gas stations (n=20) 23

Pharmacies (n=35) 24

Visit duration: data summary 25

Descriptive statistics 26

Food supermarkets 26

Shopping centres (lower limit of the range) 27

Shopping centres (upper limit of the range) 28

Coffee shops (lower limit of the range) 29

Coffee shops (upper limit of the range) 30

Fast-food restaurants (lower limit of the range) 31

Fast-food restaurants (upper limit of the range) 32

Pubs (lower limit of the range) 33

Pubs (upper limit of the range) 34

Pizza restaurants (lower limit of the range) 35

Pizza restaurants (upper limit of the range) 36

Fine dining restaurants (lower limit of the range) 37

Fine dining restaurants (upper limit of the range) 38

Retail shops non-food (lower limit of the range) 39
Retail shops non-food (upper limit of the range) 40
Gyms (lower limit of the range) 41
Gyms (upper limit of the range) 42
Hair saloons (lower limit of the range) 43
Hair saloons (upper limit of the range) 44
Banks (lower limit of the range) 45
Banks (upper limit of the range) 46
Post offices (lower limit of the range) 47
Post offices (upper limit of the range) 48
Pharmacies 49
Gas stations 50

Definition of close contact risk of Covid-19

The risk of getting COVID-19 is evolving daily and varies between and within communities. Spread happens when an infected person coughs, sneezes, or talks, and droplets from their mouth or nose are launched into the air and land in the mouths or noses of people nearby. The droplets can also be inhaled into the lungs

COVID-19 spreads mainly among people who are in close contact [1].

In our research, we used the CDC's definition of close contact, revised on October 21st, 2020:

"Close Contact: someone who was within 6 feet of an infected person for a cumulative total of 15 minutes or more over a 24-hour period* starting from 2 days before illness onset (or, for asymptomatic patients, 2 days prior to test specimen collection) until the time the patient is isolated." [2]

** Individual exposures added together over a 24-hour period (e.g., three 5-minute exposures for a total of 15 minutes). Data are limited, making it difficult to precisely define "close contact;" however, 15 cumulative minutes of exposure at a distance of 6 feet or less can be used as an operational definition for contact investigation. Factors to consider when defining close contact include proximity (closer distance likely increases exposure risk), the duration of exposure (longer exposure time likely increases exposure risk), whether the infected individual has symptoms (the period around onset of symptoms is associated with the highest levels of viral shedding), if the infected person was likely to generate respiratory aerosols (e.g., was coughing, singing, shouting), and other environmental factors (crowding, adequacy of ventilation, whether exposure was indoors or outdoors). Because the general public has not received training on proper selection and use of respiratory PPE, such as an N95, the determination of close contact should generally be made irrespective of whether the contact was wearing respiratory PPE. At this time, differential determination of close contact for those using fabric face coverings is not recommended.*

From the definition of closed contact we derived the working definition of ***closed contact risk of Covid-19*** for retail activities:

$$\frac{\text{Crowding rate/square meter}}{\text{10.4 square meters*}} \quad x \quad \frac{\text{median visit duration (minutes)}}{\text{15 minutes}}$$

* Equivalent to the area of a circle of 6 feet (1.82 cm) radius

References:

[1] Centers for Disease Control and Prevention (CDC). Social distancing. Available online at: https://www.cdc.gov/coronavirus/2019-ncov/prevent-getting-sick/social-distancing.html . Last accessed on 21/12/2020

[2] Centers for Disease Control and Prevention (CDC). Appendix A – Glossary of Key Terms. Available online at: https://www.cdc.gov/coronavirus/2019-ncov/php/contact-tracing/contact-tracing-plan/appendix.html#contact Last accessed on 21/12/2020

Maximum crowding standard by retail activity

UNI 10339 norm.

The UNI 10339 standard applies to all aeraulic systems intended for the well-being of people, installed in buildings residential and non-residential intended to control thermal, hygrometric, quality and air movement in indoor environments [3]
Appendix A, with reference to various categories of buildings, provides the values of the crowding indices (i.e. the number of people present, for design purposes, for each square meter of floor area). The crowding rates are intended to be conventional. They are fixed only for rooms where people are expected to stay.

DPCM anti Covid-19

In order to guarantee the resumption of activities, following the lockdown phase, compatibly with the trend of the epidemic, as well as the protection of the health of the staff and users, a gradual and progressive remodelling of the containment measures was necessary.

As a consequence, the Prime Ministerial Decree (DPCM) of April 26, 2020, defined that the maximum crowding standard attributed to commercial establishments was 13.3 m^2 per person (example: 3 people can enter a 40 m^2 room). The text of the Decree reads verbatim: "For premises up to forty square meters, one person can access at a time, in addition to a maximum of two operators" (i.e. 40 sqm / 3 = 13.3 sqm). [4]

In May 2020, the National Institute for Occupational Accident Insurance (INAIL) produced a technical document in collaboration with the Istituto Superiore di Sanità (ISS) with the aim of providing technical evaluation elements to the political decision-maker about the possible remodelling of the measures to contain the infection from Covid-19, with the aim of guaranteeing the health and safety of both operators and consumers. INAIL published a technical document dedicated to coffee shops and restaurants, in which it established the standard of 4 square meters per person. [5]

The following Table reports the maximum crowding by retail activity allowed by both standards: UNI (before Covid-19) and the DPCM dated April 26,2020 (after Covid-19).

RETAIL ACTIVITIES	Crowding (people in the contact area)	
	Max crowding standard (people per square meter) UNI10339, October 2008	Max crowding standard (people per square meter) DPCM anti Covid-19, April 2020
**Fine-dining restaurants**	0.66	0.250
**Pizza restaurants**	0.66	0.250
**Pubs**	0.66	0.250
**Fast-food restaurants**	0.66	0.250
**Coffee shops**	0.80	0.250
**Gyms**	0.25	0.250
**Hair salons**	0.20	0.200
**Shopping centres**	0.20	0.200
**Retail shops (non-food)**	0.20	0.075
**Food supermarkets ***	0.20	0.075
**Pharmacies ***	0.20	0.075
**Banks**	0.20	0.075
**Post offices**	0.20	0.075
**Gas stations *, ****	0.20	0.075

** Google Maps only reported the average visit duration.*

*** Max crowding standard refers to retail premises of the gas station (convenience store)*

References:

[3] Standard UNI 10339. Appendix A. Version updated on 17/10/2008. Available online at: http://www.ctslab.eu/doc/Revisione_UNI_10339_del_081017.pdf Last accessed on 21/12/2020

[4] Gazzetta Ufficiale. Prime Ministerial Decree (DPCM) of April 26, 2020. Available online at: https://www.gazzettaufficiale.it/eli/id/2020/04/27/20A02352/sg Last accessed on 11/01/2021

[5] National Institute for Occupational Accident Insurance (INAIL). Documento tecnico su ipotesi di rimodulazione delle misure contenitive del contagio da SARS-CoV-2 nel settore della ristorazione. May, 2020. Available online at: https://marcigaglia.it/wp-content/uploads/2020/05/Documento-tecnico-ristorazione-2-compresso.pdf Last accessed on 22/12/2020.

Mean visit duration in retail shopping premises: the Google Maps data

Visit duration

Since October 2020, Google made visit duration time available on Google Maps (mobile version only). This data shows how much time customers typically spend in a specific store. Visit duration estimates are based on patterns of customer visits over the past several weeks.
Visit duration is expressed in units of time (minutes ad hours)
Most retail stores show the visit duration as a range (e.g. 1.5 – 3 hours), while food supermarkets indicate a mean value (e.g. 20 minutes).

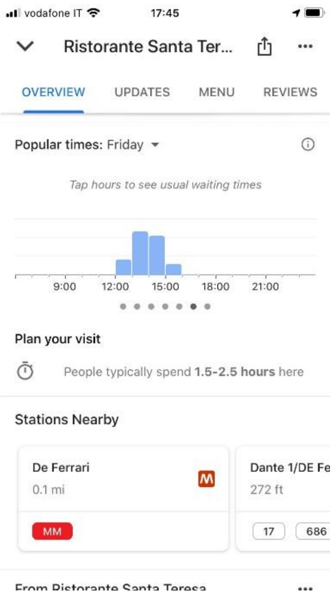

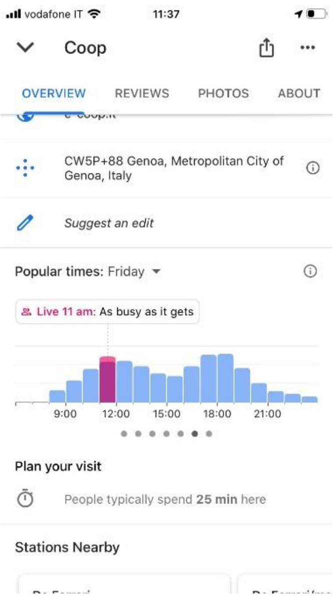

We manually collected visit duration data for 561 retail activities, banks and public offices located by Google Maps in the metropolitan area of Genoa, Italy.
The sample of visit data available included several retail activities:

Food supermarkets	n=170
Shopping centres	n= 16
Coffee shops	n= 14
Fast-food restaurants	n= 19
Pubs	n= 22
Pizza restaurants	n= 41
Fine-dining restaurants	n= 39
Retail shops (non-food)	n= 86
Gyms	n= 10
Hair salons	n= 14
Banks	n= 38
Pharmacies	n= 35
Post offices	n= 37
Gas stations	n= 20

Food supermarkets (n=170)

Store	Mean time in store (minutes)
Coop Piccapietra	25
Coop Piazzale Traghetti	25
Coop Stazione Casella	20
Coop Salita F Da Paola	20
Coop Cso Gastaldi	30
Coop Via Romairone	10
Coop Pzza Tre Ponti	25
Coop Via del Mirto	20
Coop Via Fumara	20
Coop Via Rivarolo	20
Coop Via dei Mille	20
Coop il Gabbiano	15
Coop Via Montanaro	5
Coop Cso Europa	25
Coop Via Merano	25
Coop Via Franceschi	25
Coop Viale Milite Ignoto	25
Coop LungoBisagno	20
Coop Via Roana	20
Coop Via Pra	20
Coop via del Commercio	25
Conad Via Corsica	25
Conad Cso Sardegna	20
Conad Pozza Carloforte	20
Conad Via Piccone	20
Conad Via Fiumra	10
Conad Via Trossarelli	20
Conad Via Mascagni	25
Conad Via Linneo	15
Carrefour Via delle Bernardine	20
Carrefour Via Bixio	15
Carrefour Via Casaregis	15
Carrefour Via Cesarea	25
Carrefour Pzza Merani	15
Carrefour Via Canevari	15
Carrefour Via S Vincenzo	15
Carrefour Via Fiasella	15
Carrefour Campetto	15
Carrefour Via Caffaro	15
Carrefour Via di Canneto	20
Carrefour Via S Agnese	20
Carrefour Pzza Villa	15
Carrefour Via Cecchi	15
Carrefour Vico Casana	15
Carrefour Via Albaro	15
Carrefour Via Contubernio	15
Carrefour Pzzle Parenzo	15
Carrefour Via Filzi	20
Carrefour Cso Firenze	15

Store	Mean time in store (minutes)
Carrefour Via Tortosa	20
Carrefour Via Cantore	15
Carrefour Passo Centurione	15
Carrefour Via Fareggiano	15
Carrefour Via Jori	15
Carrefour Via Bari	15
Carrefour Via Chighizola	15
Carrefour Via Rodi	15
Carrefour Via Cairoli	15
Carrefour Via Martinetti	15
Carrefour Via Fillak	20
Carrefour Via isonzo	15
Carrefour Via Rota	20
Carrefour Via S Pio X	15
Carrefour Via Fabrizi	15
Carrefour Via Quinto	20
Carrefour Via S De Rossi	15
Carrefour Via Casaregis	15
Carrefour Via Pisacane	15
Carrefour Via S Martino	15
Carrefour Via Torti	25
Carrefour Pzza Marsala	15
Carrefour Via Gobetti	15
Carrefour Via Bettini	20
PAM Via Manuzio	25
PAM Via Galata	15
PAM Via del Legaccio	45
PAM Via Cantore	15
PAM Via Chiaravagna	25
PAM Via Fiasella	20
PAM Via Verità	45
Basko S Fruttuoso	25
Basko Via barabino	20
Basko Passo Antiochia	20
Basko Via Centuriona	25
Baski Pzza Sopranis	25
Basko Via Langustena	25
Basko Via Cavallotti	25
Basko Via Sturla	20
Basko Via Posalunga	15
Basko Via Degola	20
Basko Via faliero	25
Basko Via Emilia	25
Basko Via Bertolotti	30
Basko Via Barchetta	25
Basko Via Molassana	30
Basko Via Travi	20
Basko Via Martiri Libertà	25
Basko Via Borzoli	20
Basko Via Ricasoli	15

Store	Mean time in store (minutes)
Bsko Via Anfossi	20
Basko Via Taggia	25
Basko Via Aurelia	20
Basko Via Cavour	25
Basko Via Suardi	20
Dipiù Via Alessi	20
Dipiù sampierdarena	20
Dipiù Via Borgoratti	20
Dipiù Via Borzoli	15
Dipiù Via Murtola	20
Ekom Via Colombo	20
Ekom Via Turati	15
Ekom Via De Gaspari	15
Ekom Via Donghi	20
Ekom Via Archimede	20
Ekom Via Rota	20
Ekom Via Spinola	20
Ekom Via Ponte Morosini	20
Ekom Via S Martino	15
Ekom Via Bobbio	20
Ekom albaro	20
Ekom Via del Lagaccio	15
Ekom Piazza Petrella	20
Ekom Via Passaggi	20
Ekom Via Montebruno	20
Ekom Via Ferrara	25
Ekom isonzo	20
Ekom Via Molassana	20
Ekom Via Crimea	15
ekom Via Robino	20
Ekom Via Carzino	20
Ekom Via Chiaravagna	20
Ekom Via Giovanni battista	20
Ekom Via Piacenza	15
Ekom Via Torti	15
Ekom Via Borgoratti	15
Ekom Via Marussig	20
Ekom Via Oberdan	20
Ekom Via Prà	25
Ekom Via Bysshe	20
Ekom Via della Castagna	20
Ekom Via Pineti	15
Ekom Via Camozzini	20
Doro Cso Armellini	25
Doro Passo barsanti	15
Doro Via melegari	20
Doro Via carrea	20
Doro Cso Martinetti	15
Doro Vle Franchini	15
Doro Via Bracelli	15

**Store**	**Mean time in store (minutes)**
Doro Via del Commercio	15
iN's Mura di s Chiara	20
iN's Via Cantore	15
iN's Pzza Giusti	20
iN's Via Buranello	20
iN's Via Piacenza	20
iN's Via Storace	15
iN's Via Malfettani	20
iN's Via galata	20
iN' Cso perrone	20
iN's Via Jori	20
iN's Via Casaregis	20
iN's Via Donghi	15
iN's Via Monticelli	25
iN's Via Paggi	20
iN's Via Mandoli	15
iN's Via Centurione	25
iN's Via S Martino	20
iN's Cso de Stefanis	20
iN's Pzza Caroli	15
iN's Via Parodi	25

Shopping centres (n=16)

Store	Mean time in the store (minutes)	
	Lower limit	Upper limit
Centro commerciale L'aquilone	30	90
Centro commerciale Europa	20	60
Il Terminal	25	90
Centro Commerciale Bisagno	20	60
Fiumara Shopping	10	60
Sotoripa	20	90
Shopping Center Molo 8.44	30	90
Centro commerciale Le Serre	25	60
Centro Commerciale Gabbiano	30	90
Centro Commerciale Le Lampare	15	45
I Leudi	20	60
Corte di Mare	20	45
La città sul Mare	20	60
La Riviera Shopville	30	90
Le Officine	20	60
Belforte	20	60

Coffee shops (n=14)

Store	Mean time in the store (minutes)	
	Lower limit	Upper limit
Merdiana caffè	25	60
Cafhein	25	60
Mangini	10	45
Tazze Pazze	30	90
Caffetteria degli orefici	25	60
Caffè Fogliotti	15	
Caffetteria S Giorgio	20	60
De Stefanis	15	45
Caffetteria Carignano	15	
Caffetteria S Giorgio torti	20	
Caffè degli Specchi	25	60
Caffetteria Garrè	10	45
Caffetteria dell'Oratorio	15	
Caffetteria 68R	15	45

Fast-food restaurants (n=19)

Store	Mean time in the store (minutes)	
	Lower limit	Upper limit
McDonald Via XX Settembre	20	45
Via Fiume	15	45
Via di Sottoripa	25	60
Via della Chiappella	15	
Via Mantovani	30	
Burger King Porto Antico	30	60
La Piadineria	15	30
Chicken & Chicken	15	45
Strike	45	
KFC	45	
KFC Fiumara	10	45
Star bangla	20	45
Il Masetto	20	60
EatItaly	30	90
La Moucca	30	90
Hamboo	45	
La focacceria	25	
Sapori di Genova	30	
Sbrano	15	

Pubs (n=22)

Store	Mean time in the store (minutes)	
	Lower limit	Upper limit
Scurreria	60	120
La Pinta	60	150
Birreria Imbarco 1	60	120
Old Troll	60	150
La Goletta	90	150
Il Galeone	45	120
Tartan Pub	60	150
Il Cantinone	60	120
Alle Volte	45	120
Carpe Diem	45	150
Big ben	60	150
Otium	15	90
752 Pub	60	120
Coccagna	60	150
La Goletta	45	120
Cantiere	45	120
Al Parador	30	90
Manhattan bar	30	90
Poldo	60	180
Beautiful loser	60	150
Old West	60	120
O'Connor	90	150

Pizza restaurants (n=41)

Store	Mean time in the store (minutes)	
	Lower limit	Upper limit
Corner	45	150
Da Pino	60	90
Pinsaccio	60	120
Exultate	60	120
La Sosta degli artisti	60	120
La Locanda del Molo	60	120
Europa Restaurant	45	90
Brera	45	90
Canneto	60	120
I Tre Merli	90	120
Punta Vagno	45	150
Alice	120	180
Garden	120	180
Pizza Express	90	150
Pizza del ponte	90	150
Il Baluardo	60	180
Piuma	90	150
Antola	45	90
E prie de ma	45	90
5 Maggio	90	150
Sole Luna	90	150
Rosso Pomodoro	20	120
Il Vicolo	60	150
Sereno 1950	60	150
Rosso pomodoro Porto Antico	90	150
Moa	45	120
La Tana del Gusto	60	120
Pestello d'oro	60	120
La Ola	90	150
MoroMare	45	120
Massarjia	45	90
Al Pisacane	60	120
Sugo	60	120
PizzaMaria	60	120
Ferro & Fuoco	45	150
Va Pensiero	90	150
La Superba	60	120
Pinseria	60	120
Tifils	60	150
Al solito posto	45	150
Osteria della Piazza	60	120

Fine dining restaurants (n=39)

Store	Mean time in the store (minutes)	
	Lower limit	Upper limit
Soho	60	120
Da Genio	60	120
Da Rina		120
Ippogrifo	60	120
Il Genovese	60	120
Pintor1	90	150
Pesciolino	90	150
Osteria Vico Palla	90	150
Trattoria Ugo	60	120
Vejai Zena	90	150
Alle Due Torri	60	120
Le Colonne		120
Da Maria	45	150
Yakido	60	120
Trattoria Osvaldo	90	180
Ostajetta	60	150
Rosmarino		90
Ittiturismo	90	150
Punta Tre Pini	90	180
Le Rune	90	150
Officina di Cucina	90	150
I Tre merli	90	120
Forchetta curiosa	60	150
La Perla	60	150
Settepolpette	60	120
Trallallero	60	120
Perlage	90	150
Le Cicale	60	120
Rustichello	60	120
Da Gibba	90	150
Osteria sopra Il mare	90	150
Mangiabuono	60	120
Santa teresa	90	150
Il Gelsomino	90	180
Cavour Modo 21	60	120
Raibetta	60	120
Da marcello	45	150
Ostaja S Vincenzo	30	60
20 Tre Ristorante	90	150

Retail shops non food (n=86)

Store	Mean time in the store (minutes)	
	Lower limit	Upper limit
Decathlon		30
Dainese		25
Unieuro		25
Lego store		20
Motivi	20	45
Sun City		25
Tigotà		20
Dmail		25
Stradivarius		25
Ghiglino		25
Intrend	30	90
Tessil Moda		20
Calzedonia		25
Wheelup		25
MD Market		25
Game People		20
H&M		30
Moisman Sport		25
Borotalco		25
Salewa		25
Smart Phones	15	45
MediaWorld		15
Papillon		30
Geox		30
Tipinifini	25	60
Piana utensili		15
Bata		25
Bricoman	20	45
Nespresso		20
Kasanova		25
Foot Locker		25
Nerska		30
Bikers store		25
Brandy Melville		20
Benetton		25
Mondadori bookstore		30
Supershop		20
Sampdoria point		20
Comics corner		30
Thun shop		20

Store	Mean time in the store (minutes)	
	Lower limit	Upper limit
Tigotà		20
Leroy Merlin	25	60
420 shop		20
Dungeon		20
Motivi		10
Globo	30	60
Il libraccio		30
Promoclub		30
Valigeria Sanson		25
Samsonite	15	45
Acqua & Sapone		20
Flying tiger		25
Mi store		10
Antony Morato		30
Primadonna		30
Scarpe & scarpe		15
Boggi	15	45
Maison du Monde		30
Zuicki		10
Pitta Rosso		10
Wurth		15
Arredo 3 store	20	90
COIN	20	45
Guess		10
Ferramenta Fabio		10
Bag store		10
QVS		15
Subdued		25
Toys centre		30
La formica econegozio		20
Lush		20
Triumph lingerie		30
Fisherlandia		20
Equivalenza		25
Deichman		30
Bassetti		25
Asta del Mobile	20	45
Bimbostore		25
Expert		20
La Befana		15
Peter Tea House		25
Stroili		30
SAGE sanitaria		20

Store	Mean time in the store (minutes)	
	Lower limit	Upper limit
Primo		20
METRO	20	60
Feltrinelli book & Music		45

Gyms (n=10)

Store	Mean time in the store (minutes)	
	Lower limit	Upper limit
New Gold Gym	60	120
Area 51	60	120
Well & Fit	5	45
CrossFit	60	120
Fit Club	60	120
Art's school	20	90
TNT fitness center	25	90
Gli Sportivi	90	150
Palestra Empire	20	90
Transition Performance	45	120

Hair saloons (n=14)

Store	Mean time in the store (minutes)	
	Lower limit	Upper limit
Billion Hair	45	120
TJ Favour John	30	60
JL David	45	150
Equipe Giorgio Via XX Settembre	45	90
Top Hair salon	45	120
Hairteca	25	60
Luca & Manola hairstyle	30	90
Nadege Hairdresser	10	60
Equipe Giorgio Lab Haidresser	45	150
Geko	30	90
G&Y Parrucchieri	30	120
Centro Degradè	60	180
Elisa Look & Style	15	60
Le Figarò Hairdresser	25	60

Post offices (n=57)

Store	Mean time in the store (minutes)	
	Lower limit	Upper limit
Poste Via Dante	20	45
Poste Via Granello	20	
Poste Via Ilva	15	
Poste Piazza Rovere	20	
Poste Via valle Chiara	15	45
Poste Via Pozzo	20	
Poste Via Canevari	15	
Poste Via Francia	15	45
Poste Via san Fruttuoso	25	
Poste Via Colombo	20	
Poste Via Assarotti	20	
Poste Cso Sardegna	20	
Poste Piazza Monastero	20	60
Poste Via Pisa	15	
Poste Piazzale Marassi	25	
Poste Piazza Acquaverde	15	45
Poste Via Langustena	15	
Poste Via S Franceco de Paola	15	45
Poste Cso Europa	15	
Poste Cso Marconi	15	
Poste Via Donghi	15	
Poste Via Fereggiano	20	
Poste Cso Europa	15	
Poste Cso sardegna	20	
Poste Via Martinetti	20	
Poste Via Re di Puglia	25	
Poste Piazzale Sigelli	15	
Poste Via Ulanowski	10	45
Poste Via Cembrano	15	
Poste Via Carbone	10	45
Poste Via Blelè	15	
Poste Via Piacenza	15	
Poste Via Robino	15	
Poste Via Roggerone	20	
Poste Via Terpi	20	
Poste Via Catalai	25	
Poste Via Giannelli	15	
Poste Via Schiaffino	15	45
Poste Via Struppa	15	
Poste Via Molassana	20	

Store	Mean time in the store (minutes)	
	Lower limit	Upper limit
Poste Via Pastorino	20	
Poste Via martiri della Libertà	20	45
Poste Via Franchini	20	
Poste Via Caldesi	20	
Poste Via Orsini	15	
Poste Via Olivieri	15	
Poste Via da Pozzo	20	
Poste Via Toscanelli	25	
Poste Via Piccone	15	45
Poste Via dal Canto	20	
Poste Via Airaghi	20	
Poste Via Multedo	15	
Poste Piazza Gaggero	20	
Poste Via Borzoli	15	
Poste Via San Quirico	20	
Poste Via Spalato	15	
Poste Via San Romolo	15	

Banks (n=38)

Store	Mean time in the store (minutes)	
	Lower limit	Upper limit
Banca Sella Via Fieschi	20	
BNL Via Brigata Liguria	15	
Banca CARIGE Via Corsica	10	45
Banca Italia Via Dante	20	90
Unicredit Via Fiasella	15	45
Banca di Asti Via Brigata Liguria	15	
CheBanca! Via Roma	10	45
Unicredit Via Garibaldi	20	
BNL Largo Lanfranco	15	60
Credem Piazza Dante	25	
Intesa Pzza Fontane Marose	15	
BNL Corso Torino	10	45
Banco di Sardegna Pzza Fontane Marose	25	
BPER Banca Viale Brigate Bisagno	10	45
Credit Agricole Via XX Settembre	15	45
UBI Banca Via Ceccardi	15	45
Banca CARIGE Cso Firenze	20	
Unicredit Via Brigate Partigiane	15	
Banca Credito Cooperativo di Cherasco	15	45
CheBanca! Genova Bisagno	10	45
Deutsche Bank Via Fieschi	20	
Banca di Sondrio Via XXV Aprile	25	
Intesa Via Cecchi	20	
Carige Via Libertà	20	
ING Pzza Dante	15	
BNL Via Torti	15	45
Intesa Via Fieschi	15	45
Unicredit Cso Benos aires	20	
Carige Pzza Leopardi	15	
Banca di Sondrio Pzza Tomasseo	15	
Credito su Pegno	30	
Unicredit Via Gastaldi	10	
Intesa Cso firenze	10	45
Intesa Cso sardegna	15	
Unicredit Via Vittorio veneto	15	
Unicredit Via Piacenza	15	
Intesa Via Gioacchino Rossini	15	
Intesa Via Timavo	15	

Gas stations (n=20)

Store	Mean time in the store (minutes)
Q8 Genova Nervi	15
Esso Genova Piazza Dinegro	10
Bolzaneto Station Service	10
Esso PiazzaManin	10
Europam Via Bianchi	10
IP Via Diaz	10
Europam Cso Magenta	10
Esso Cso Europa	10
Q8 Lanterna Est	10
Q8 Piazza Brignole	10
Q8 Cso Sardegna	10
Europam Via Barzoli	10
ESSO Cornigliano	10
Metano Via Piacenza	10
Tamoil Va Canepari	10
Autoservice Cso Europa	15
Q8 Via Barzoli	10
Automobilgas Via Borzoli	10
IP Via Le Merle	10
Europam Via Marconi	10

Pharmacies (n=35)

Store	Mean time in the store (minutes)
Farmacia Alvigini	15
Farmacia Papa	15
Farmacia pescetto	10
Farmacia Barabino	15
Farmacia Bassano	20
Farmacia Nizza	15
Farmacia Del Chiappazzo	15
Farmacia Saltarelli	20
Framacia S Giacomo	15
Farmacia Giusto	15
Farmacia Nazionale	15
Farmacia Assarotti	10
Farmacia Bonanni	15
Farmacia Ghersi	15
Farmacia Monticelli	15
Farmacia Castelletto	15
Farmacia Ponte Monumentale	15
Farmacia Ribaldone	10
Farmacia del Castello	15
Farmacia del Porto	10
Farmacia de Ferrari	10
Farmacia della Marina	15
Farmacia Canevari	15
Farmacia Martinelli	15
Farmacia Spelta	15
Framacia Modigliani	15
Farmacia Piaggio	15
Farmacia San Raffaele	10
Farmacia Caprera	15
Farmacia Centrale	15
Farmacia san Tommaso	10
Farmacia Palmaro	15
Farmacia Sestri	15
Farmacia iachetti	15
Farmacia Negrotto	15

Visit duration: data summary

RETAIL ACTIVITIES	Visit duration by retail activity in the metropolitan area of Genoa, Italy (source: Google Maps, 30/12/2020)							
	Lower limit of the range				Upper limit of the range			
	Sample (n)	Median visit duration (minutes)	95% Confidence Interval of the median		Sample (n)	Median visit duration (minutes)	95% Confidence Interval of the median	
**Pubs**	22	60.0	45.0	60.0	22	120.0	120.0	150.0
**Pizza restaurants**	41	60.0	60.0	60.0	41	120.0	120.0	150.0
**Fine-dining restaurants**	36	60.0	60.0	90.0	39	150.0	120.0	150.0
**Gyms**	10	52.5	20.0	60.0	10	120.0	90.0	120.0
**Hair salons**	14	30.0	25.0	45.0	14	90.0	60.0	123.1
**Fast-food restaurants**	19	25.0	15.0	30.0	11	45.0	45.0	65.4
**Food supermarkets ***	170	20.0	20.0	20.0	N/A	N/A	N/A	N/A
**Shopping centres**	16	20.0	20.0	26.8	16	60.0	60.0	90.0
**Retail shops (non-food)**	13	20.0	17.7	25.0	86	25.0	25.0	25.0
**Coffee shops**	14	17.5	15.0	25.0	10	60.0	45.0	60.0
**Banks**	38	15.0	15.0	15.0	14	45.0	45.0	45.0
**Pharmacies ***	35	15.0	15.0	15.0	N/A	N/A	N/A	N/A
**Post offices**	57	15.0	15.0	20.0	11	45.0	45.0	45.0
**Gas stations *,****	20	10.0	10.0	10.0	N/A	N/A	N/A	N/A

** Google Maps only reported the average visit duration.*

*** Max crowding standard refers to retail premises of the gas station (convenience store)*

Descriptive statistics

Statistical software: MedCalc

Food supermarkets

Sample size	170
Lowest value	5.0000
Highest value	45.0000
Arithmetic mean	19.4412
95% CI for the Arithmetic mean	18.6889 to 20.1934
Median	20.0000
95% CI for the median	20.0000 to 20.0000
Variance	24.6859
Standard deviation	4.9685
Relative standard deviation	0.2556 (25.56%)
Standard error of the mean	0.3811
Coefficient of Skewness	1.5816 (P<0.0001)
Coefficient of Kurtosis	7.0342 (P<0.0001)
Shapiro-Wilk test for Normal distribution	W=0.8008 reject Normality (P<0.0001)

Percentiles		95% Confidence interval
2.5	15.0000	6.5165 to 15.0000
5	15.0000	14.5926 to 15.0000
10	15.0000	15.0000 to 15.0000
25	15.0000	15.0000 to 15.0000
75	20.0000	20.0000 to 25.0000
90	25.0000	25.0000 to 25.0000
95	25.0000	25.0000 to 30.0000
97.5	30.0000	25.0000 to 45.0000

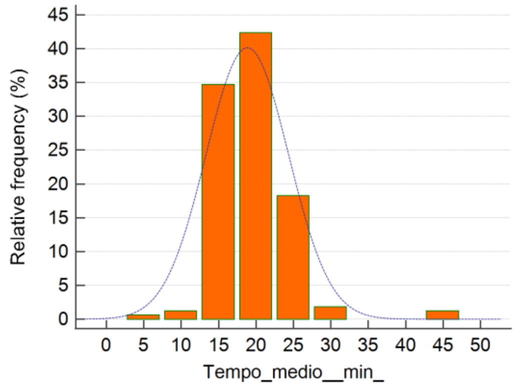

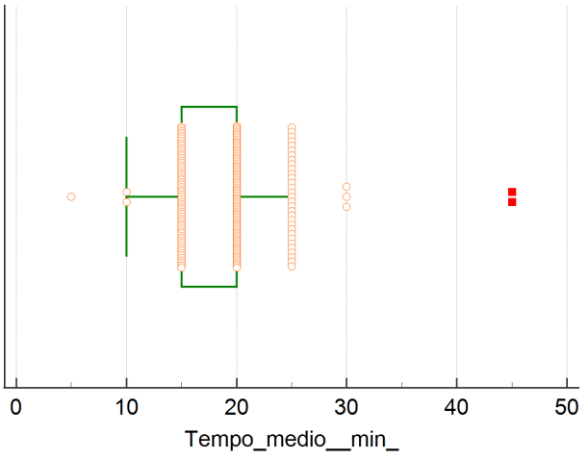

Shopping centres (lower limit of the range)

Sample size	16
Lowest value	10.0000
Highest value	30.0000
Arithmetic mean	22.1875
95% CI for the Arithmetic mean	19.1158 to 25.2592
Median	20.0000
95% CI for the median	20.0000 to 26.8074
Variance	33.2292
Standard deviation	5.7645
Relative standard deviation	0.2598 (25.98%)
Standard error of the mean	1.4411
Coefficient of Skewness	-0.1247 (P=0.8151)
Coefficient of Kurtosis	-0.1048 (P=0.9032)
Shapiro-Wilk test for Normal distribution	W=0.8625 reject Normality (P=0.0209)

Percentiles		95% Confidence interval
2.5		
5	11.5000	
10	15.5000	
25	20.0000	12.1403 to 20.0000
75	27.5000	20.0000 to 30.0000
90	30.0000	
95	30.0000	
97.5		

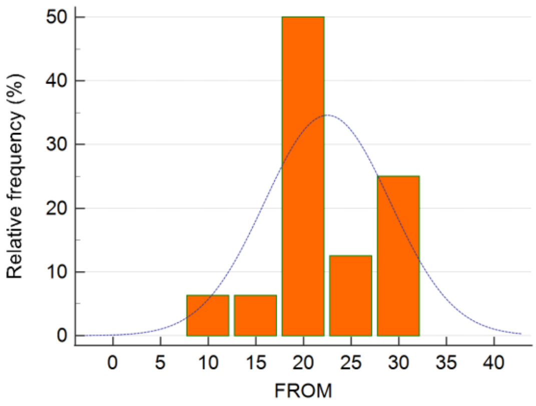

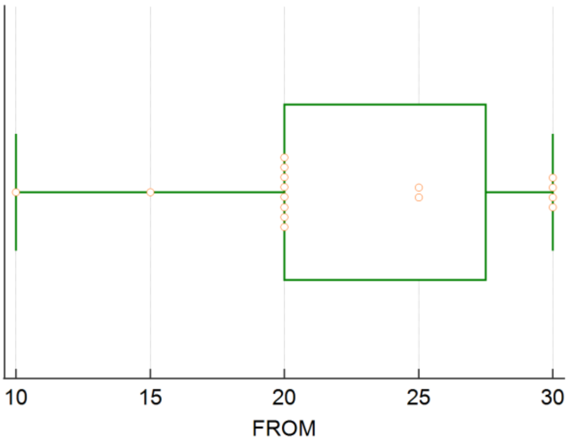

Shopping centres (upper limit of the range)

Sample size	16
Lowest value	45.0000
Highest value	90.0000
Arithmetic mean	69.3750
95% CI for the Arithmetic mean	60.2034 to 78.5466
Median	60.0000
95% CI for the median	60.0000 to 90.0000
Variance	296.2500
Standard deviation	17.2119
Relative standard deviation	0.2481 (24.81%)
Standard error of the mean	4.3030
Coefficient of Skewness	0.2553 (P=0.6332)
Coefficient of Kurtosis	-1.6046 (P=0.0174)
Shapiro-Wilk test for Normal distribution	W=0.7649 reject Normality (P=0.0010)

Percentiles		95% Confidence interval
2.5		
5	45.0000	
10	46.5000	
25	60.0000	45.0000 to 60.0000
75	90.0000	60.0000 to 90.0000
90	90.0000	
95	90.0000	
97.5		

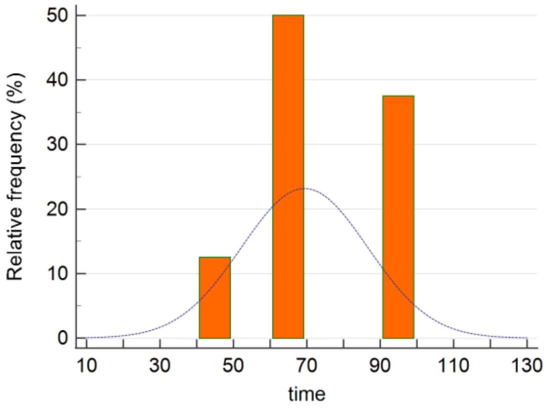

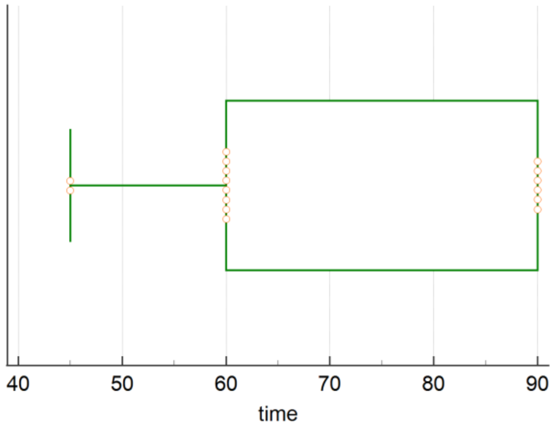

Coffee shops (lower limit of the range)

Sample size	14
Lowest value	10.0000
Highest value	30.0000
Arithmetic mean	18.9286
95% CI for the Arithmetic mean	15.3160 to 22.5412
Median	17.5000
95% CI for the median	15.0000 to 25.0000
Variance	39.1484
Standard deviation	6.2569
Relative standard deviation	0.3306 (33.06%)
Standard error of the mean	1.6722
Coefficient of Skewness	0.1935 (P=0.7330)
Coefficient of Kurtosis	-1.0969 (P=0.2626)
Shapiro-Wilk test for Normal distribution	W=0.9054 accept Normality (P=0.1351)

Percentiles		95% Confidence interval
2.5		
5	10.0000	
10	10.0000	
25	15.0000	10.0000 to 16.7292
75	25.0000	18.2708 to 29.2319
90	25.5000	
95	29.0000	
97.5		

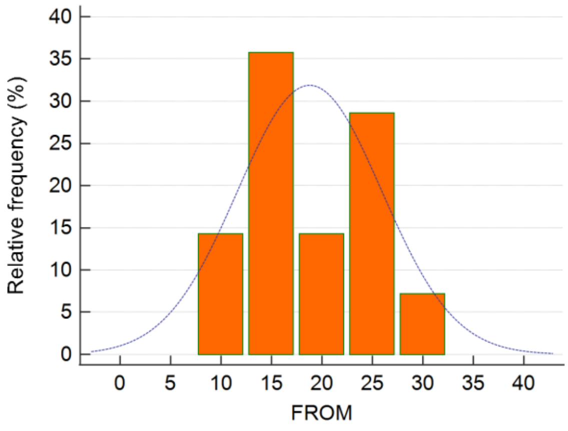

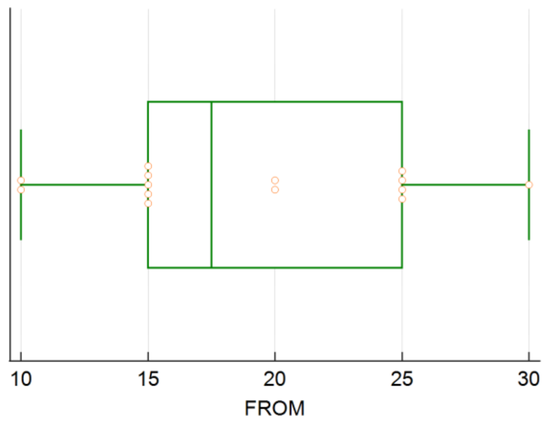

Coffee shops (upper limit of the range)

Sample size	10
Lowest value	45.0000
Highest value	90.0000
Arithmetic mean	57.0000
95% CI for the Arithmetic mean	47.1395 to 66.8605
Median	60.0000
95% CI for the median	45.0000 to 60.0000
Variance	190.0000
Standard deviation	13.7840
Relative standard deviation	0.2418 (24.18%)
Standard error of the mean	4.3589
Coefficient of Skewness	1.5464 (P=0.0275)
Coefficient of Kurtosis	3.3345 (P=0.0474)
Shapiro-Wilk test for Normal distribution	W=0.7499 reject Normality (P=0.0036)

Percentiles		95% Confidence interval
2.5		
5	45.0000	
10	45.0000	
25	45.0000	
75	60.0000	
90	75.0000	
95	90.0000	
97.5		

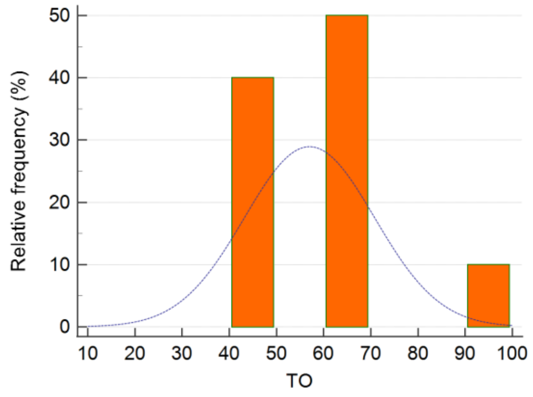

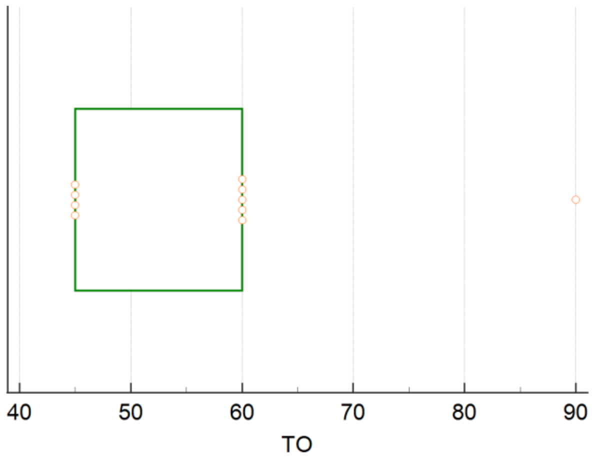

Fast-food restaurants (lower limit of the range)

Sample size	19
Lowest value	10.0000
Highest value	45.0000
Arithmetic mean	25.2632
95% CI for the Arithmetic mean	20.0279 to 30.4985
Median	25.0000
95% CI for the median	15.0000 to 30.0000
Variance	117.9825
Standard deviation	10.8620
Relative standard deviation	0.4300 (43.00%)
Standard error of the mean	2.4919
Coefficient of Skewness	0.6879 (P=0.1781)
Coefficient of Kurtosis	-0.3965 (P=0.8108)
Shapiro-Wilk test<br>for Normal distribution	W=0.8851<br>reject Normality (P=0.0263)

Percentiles		95% Confidence interval
2.5		
5	12.2500	
10	15.0000	
25	15.0000	14.3288 to 20.6393
75	30.0000	25.0000 to 45.0000
90	45.0000	
95	45.0000	
97.5		

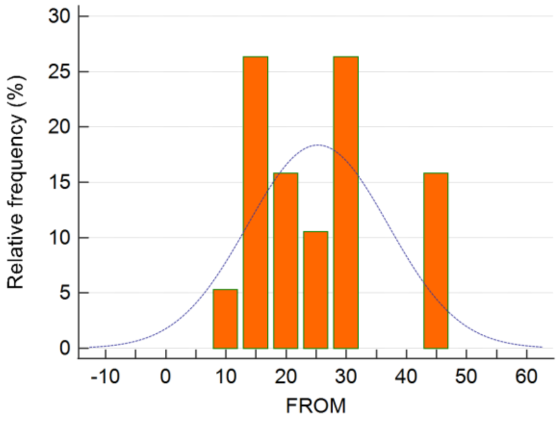

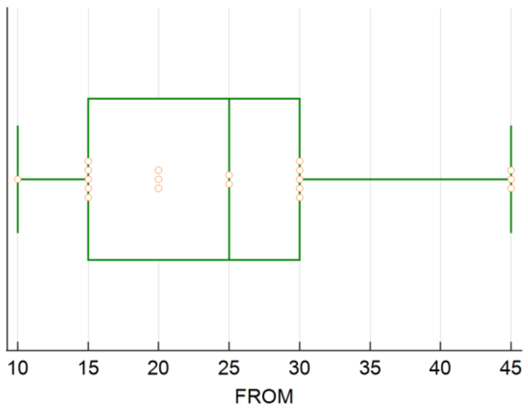

Fast-food restaurants (upper limit of the range)

Sample size	11
Lowest value	30.0000
Highest value	90.0000
Arithmetic mean	55.9091
95% CI for the Arithmetic mean	43.0902 to 68.7280
Median	45.0000
95% CI for the median	45.0000 to 65.3908
Variance	364.0909
Standard deviation	19.0812
Relative standard deviation	0.3413 (34.13%)
Standard error of the mean	5.7532
Coefficient of Skewness	0.9775 (P=0.1345)
Coefficient of Kurtosis	0.2916 (P=0.6596)
Shapiro-Wilk test for Normal distribution	W=0.8340 reject Normality (P=0.0264)

Percentiles		95% Confidence interval
2.5		
5	30.7500	
10	39.0000	
25	45.0000	
75	60.0000	
90	90.0000	
95	90.0000	
97.5		

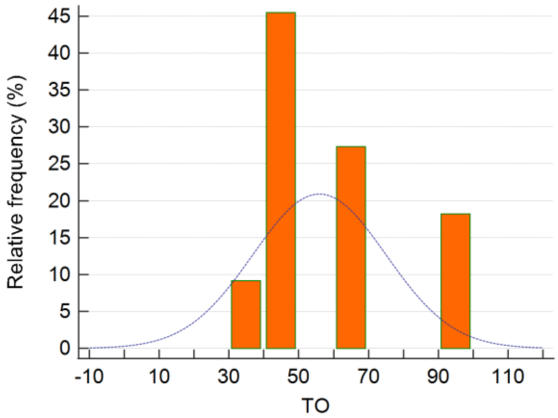

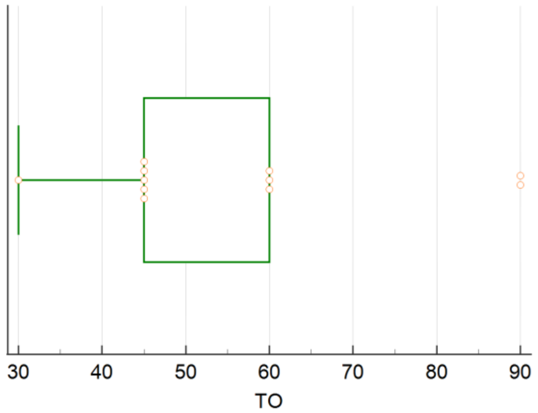

Pubs (lower limit of the range)

Sample size	22
Lowest value	15.0000
Highest value	90.0000
Arithmetic mean	54.5455
95% CI for the Arithmetic mean	46.9917 to 62.0992
Median	60.0000
95% CI for the median	45.0000 to 60.0000
Variance	290.2597
Standard deviation	17.0370
Relative standard deviation	0.3123 (31.23%)
Standard error of the mean	3.6323
Coefficient of Skewness	-0.04964 (P=0.9144)
Coefficient of Kurtosis	1.3231 (P=0.1707)
Shapiro-Wilk test for Normal distribution	W=0.8552 reject Normality (P=0.0042)

Percentiles		95% Confidence interval
2.5	15.7500	
5	24.0000	
10	30.0000	
25	45.0000	30.0000 to 60.0000
75	60.0000	60.0000 to 80.1517
90	69.0000	
95	90.0000	
97.5	90.0000	

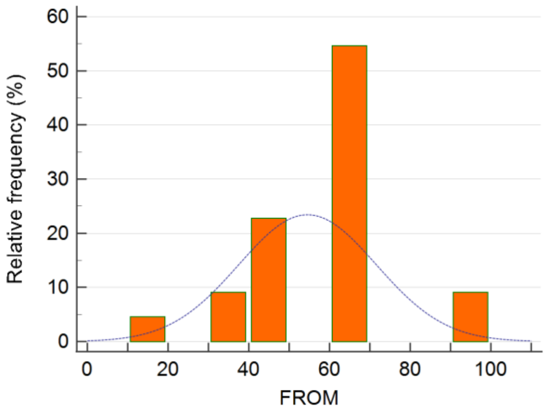

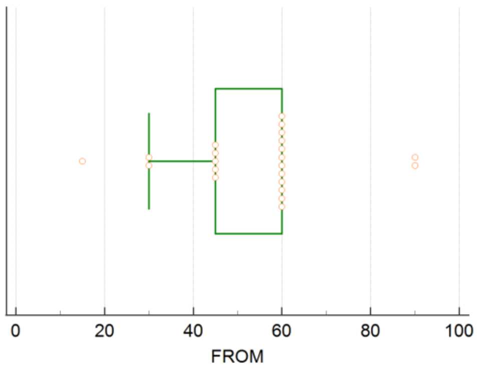

Pubs (upper limit of the range)

Sample size	22
Lowest value	90.0000
Highest value	180.0000
Arithmetic mean	130.9091
95% CI for the Arithmetic mean	120.4072 to 141.4110
Median	120.0000
95% CI for the median	120.0000 to 150.0000
Variance	561.0390
Standard deviation	23.6863
Relative standard deviation	0.1809 (18.09%)
Standard error of the mean	5.0499
Coefficient of Skewness	-0.1425 (P=0.7581)
Coefficient of Kurtosis	-0.3525 (P=0.8330)
Shapiro-Wilk test for Normal distribution	W=0.8609 reject Normality (P=0.0053)

Percentiles		95% Confidence interval
2.5	90.0000	
5	90.0000	
10	90.0000	
25	120.0000	90.0000 to 120.0000
75	150.0000	145.1150 to 150.0000
90	150.0000	
95	162.0000	
97.5	178.5000	

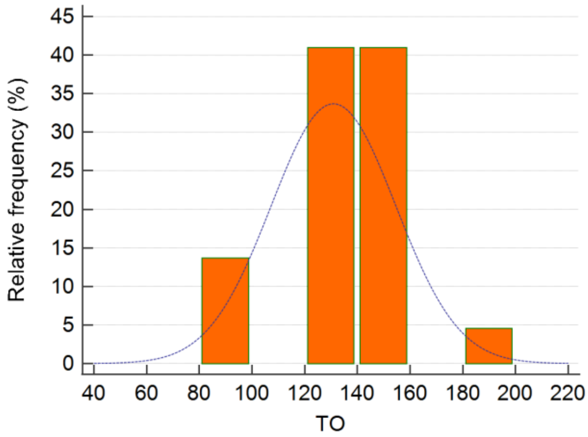

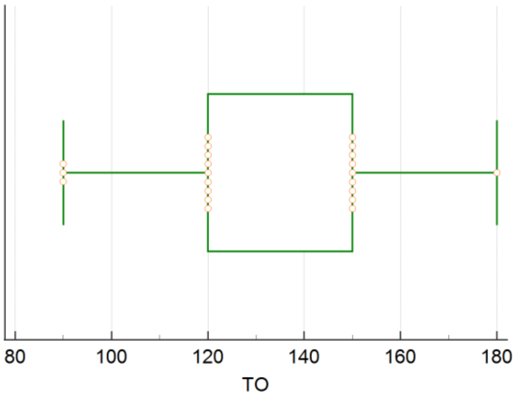

Pizza restaurants (lower limit of the range)

Sample size	41
Lowest value	20.0000
Highest value	120.0000
Arithmetic mean	64.5122
95% CI for the Arithmetic mean	57.7167 to 71.3076
Median	60.0000
95% CI for the median	60.0000 to 60.0000
Variance	463.5061
Standard deviation	21.5292
Relative standard deviation	0.3337 (33.37%)
Standard error of the mean	3.3623
Coefficient of Skewness	0.8405 (P=0.0274)
Coefficient of Kurtosis	0.5856 (P=0.3364)
Shapiro-Wilk test for Normal distribution	W=0.8487 reject Normality (P=0.0001)

Percentiles		95% Confidence interval
2.5	33.1250	
5	45.0000	
10	45.0000	27.6252 to 45.0000
25	45.0000	45.0000 to 60.0000
75	90.0000	60.0000 to 90.0000
90	90.0000	90.0000 to 120.0000
95	103.5000	
97.5	120.0000	

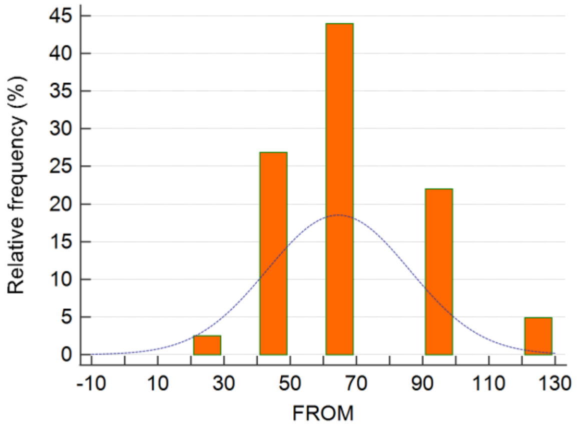

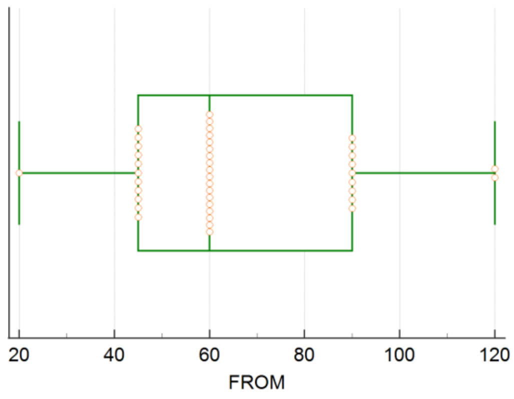

Pizza restaurants (upper limit of the range)

Sample size	41
Lowest value	90.0000
Highest value	180.0000
Arithmetic mean	130.9756
95% CI for the Arithmetic mean	123.1224 to 138.8288
Median	120.0000
95% CI for the median	120.0000 to 150.0000
Variance	619.0244
Standard deviation	24.8802
Relative standard deviation	0.1900 (19.00%)
Standard error of the mean	3.8856
Coefficient of Skewness	0.03667 (P=0.9166)
Coefficient of Kurtosis	-0.4753 (P=0.5340)
Shapiro-Wilk test for Normal distribution	W=0.8698 reject Normality (P=0.0002)

Percentiles		95% Confidence interval
2.5	90.0000	
5	90.0000	
10	90.0000	90.0000 to 120.0000
25	120.0000	90.0000 to 120.0000
75	150.0000	150.0000 to 150.0000
90	150.0000	150.0000 to 180.0000
95	180.0000	
97.5	180.0000	

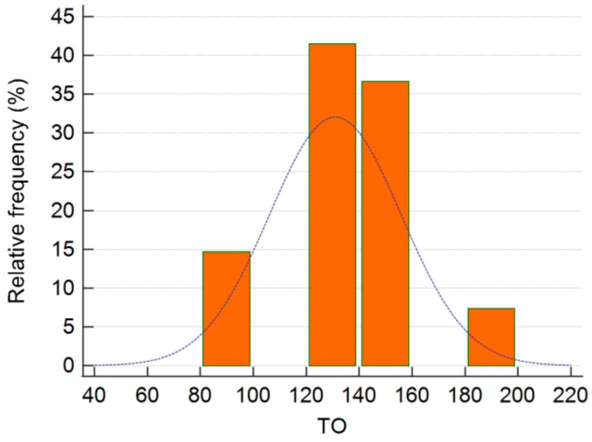

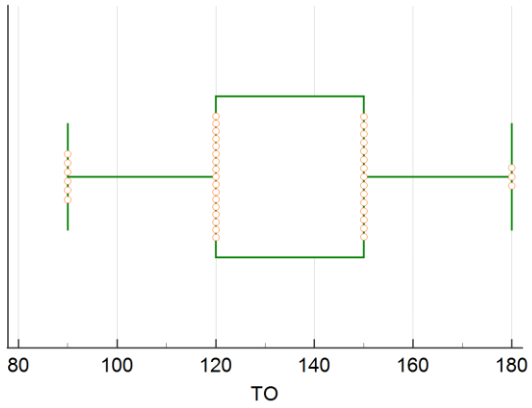

Fine dining restaurants (lower limit of the range)

Sample size	36
Lowest value	30.0000
Highest value	90.0000
Arithmetic mean	71.6667
95% CI for the Arithmetic mean	65.7094 to 77.6240
Median	60.0000
95% CI for the median	60.0000 to 90.0000
Variance	310.0000
Standard deviation	17.6068
Relative standard deviation	0.2457 (24.57%)
Standard error of the mean	2.9345
Coefficient of Skewness	-0.2143 (P=0.5658)
Coefficient of Kurtosis	-1.1055 (P=0.0221)
Shapiro-Wilk test for Normal distribution	W=0.7621 reject Normality (P<0.0001)

Percentiles		95% Confidence interval
2.5	36.0000	
5	45.0000	
10	60.0000	30.7294 to 60.0000
25	60.0000	60.0000 to 60.0000
75	90.0000	90.0000 to 90.0000
90	90.0000	90.0000 to 90.0000
95	90.0000	
97.5	90.0000	

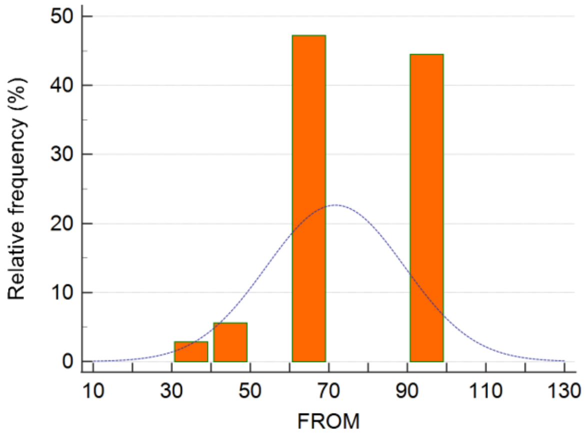

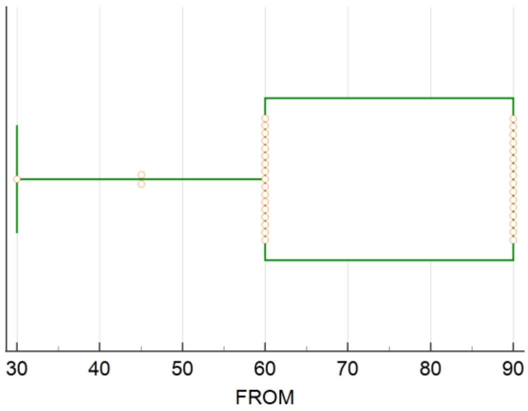

Fine dining restaurants (upper limit of the range)

Sample size	39
Lowest value	60.0000
Highest value	180.0000
Arithmetic mean	135.3846
95% CI for the Arithmetic mean	127.6975 to 143.0718
Median	150.0000
95% CI for the median	120.0000 to 150.0000
Variance	562.3482
Standard deviation	23.7139
Relative standard deviation	0.1752 (17.52%)
Standard error of the mean	3.7973
Coefficient of Skewness	-0.5497 (P=0.1397)
Coefficient of Kurtosis	1.6356 (P=0.0684)
Shapiro-Wilk test for Normal distribution	W=0.8323 reject Normality (P<0.0001)

Percentiles		95% Confidence interval
2.5	74.2500	
5	103.5000	
10	120.0000	66.0149 to 120.0000
25	120.0000	120.0000 to 120.0000
75	150.0000	150.0000 to 150.0000
90	150.0000	150.0000 to 180.0000
95	180.0000	
97.5	180.0000	

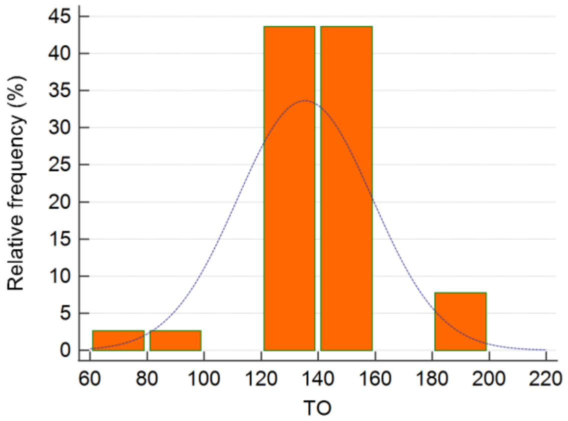

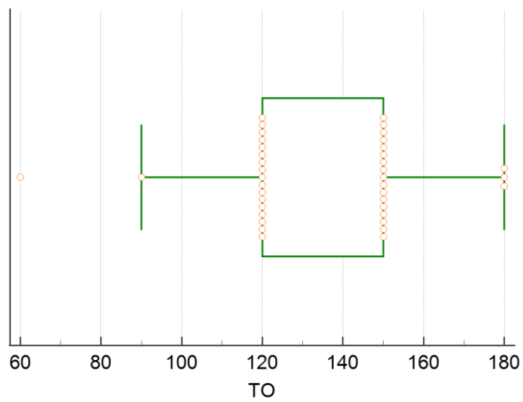

Retail shops non-food (lower limit of the range)

Sample size	13
Lowest value	15.0000
Highest value	30.0000
Arithmetic mean	21.1538
95% CI for the Arithmetic mean	18.0939 to 24.2138
Median	20.0000
95% CI for the median	17.6575 to 25.0000
Variance	25.6410
Standard deviation	5.0637
Relative standard deviation	0.2394 (23.94%)
Standard error of the mean	1.4044
Coefficient of Skewness	0.5992 (P=0.3144)
Coefficient of Kurtosis	-0.3625 (P=0.8935)
Shapiro-Wilk test for Normal distribution	W=0.8624 reject Normality (P=0.0415)

Percentiles		95% Confidence interval
2.5		
5	15.0000	
10	15.0000	
25	18.7500	15.0000 to 20.0000
75	25.0000	20.0000 to 30.0000
90	30.0000	
95	30.0000	
97.5		

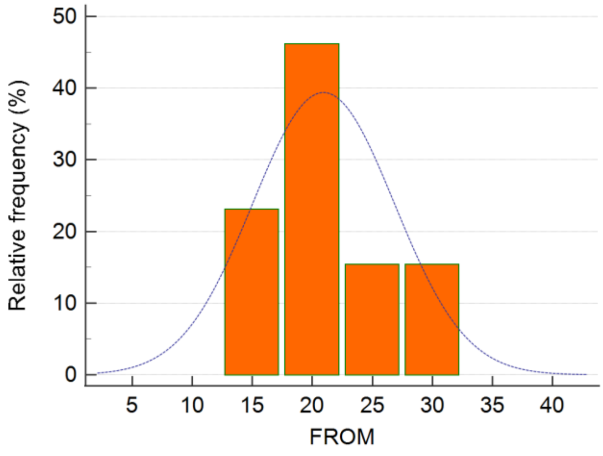

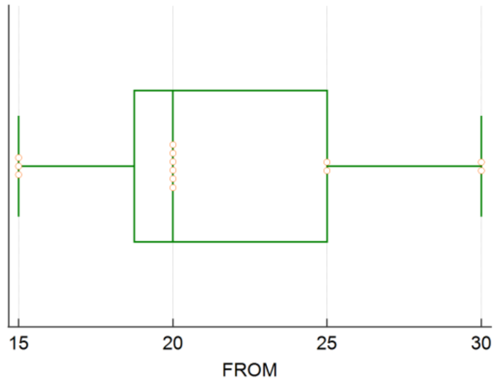

Retail shops non-food (upper limit of the range)

Sample size	86
Lowest value	10.0000
Highest value	90.0000
Arithmetic mean	27.9070
95% CI for the Arithmetic mean	24.7122 to 31.1017
Median	25.0000
95% CI for the median	25.0000 to 25.0000
Variance	222.0383
Standard deviation	14.9009
Relative standard deviation	0.5340 (53.40%)
Standard error of the mean	1.6068
Coefficient of Skewness	2.1408 (P<0.0001)
Coefficient of Kurtosis	6.0127 (P<0.0001)
Shapiro-Wilk test for Normal distribution	W=0.7748 reject Normality (P<0.0001)

Percentiles		95% Confidence interval
2.5	10.0000	
5	10.0000	10.0000 to 15.0000
10	15.0000	10.0000 to 20.0000
25	20.0000	20.0000 to 20.0000
75	30.0000	29.6111 to 38.9937
90	45.0000	31.7029 to 60.0000
95	60.0000	45.0000 to 90.0000
97.5	70.5000	

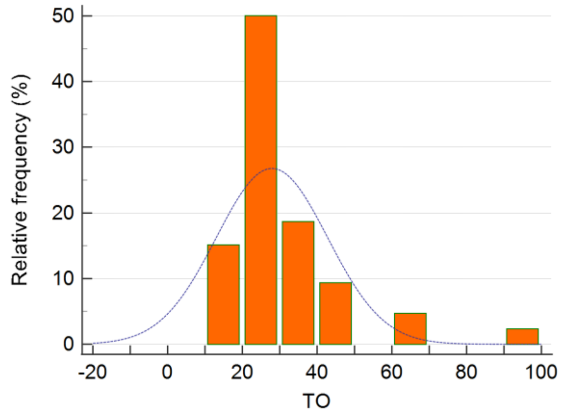

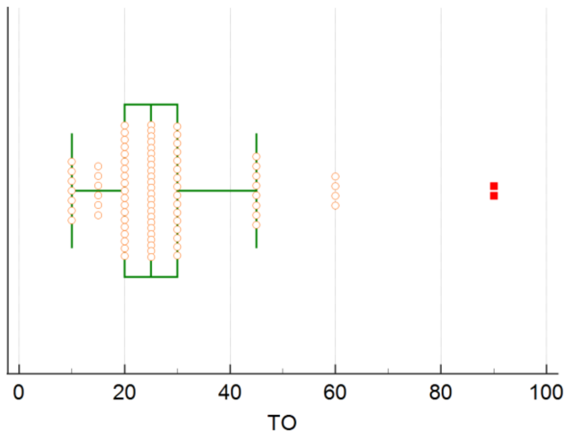

Gyms (lower limit of the range)

Sample size	10
Lowest value	5.0000
Highest value	90.0000
Arithmetic mean	44.5000
95% CI for the Arithmetic mean	25.7659 to 63.2341
Median	52.5000
95% CI for the median	20.0000 to 60.0000
Variance	685.8333
Standard deviation	26.1884
Relative standard deviation	0.5885 (58.85%)
Standard error of the mean	8.2815
Coefficient of Skewness	0.08224 (P=0.9012)
Coefficient of Kurtosis	-0.7124 (P=0.6591)
Shapiro-Wilk test for Normal distribution	W=0.9210 accept Normality (P=0.3653)

Percentiles		95% Confidence interval
2.5		
5	5.0000	
10	12.5000	
25	20.0000	
75	60.0000	
90	75.0000	
95	90.0000	
97.5		

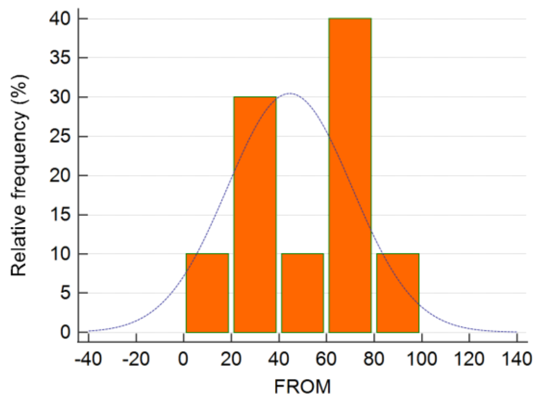

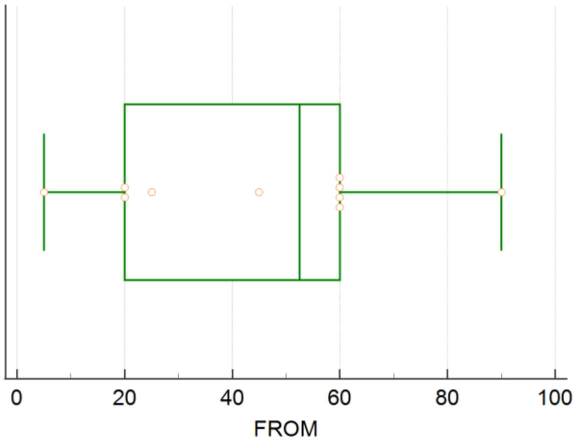

Gyms (upper limit of the range)

Sample size	10
Lowest value	45.0000
Highest value	150.0000
Arithmetic mean	106.5000
95% CI for the Arithmetic mean	85.9841 to 127.0159
Median	120.0000
95% CI for the median	90.0000 to 120.0000
Variance	822.5000
Standard deviation	28.6793
Relative standard deviation	0.2693 (26.93%)
Standard error of the mean	9.0692
Coefficient of Skewness	-0.8918 (P=0.1873)
Coefficient of Kurtosis	1.5564 (P=0.2288)
Shapiro-Wilk test for Normal distribution	W=0.8694 accept Normality (P=0.0984)

Percentiles		95% Confidence interval
2.5		
5	45.0000	
10	67.5000	
25	90.0000	
75	120.0000	
90	135.0000	
95	150.0000	
97.5		

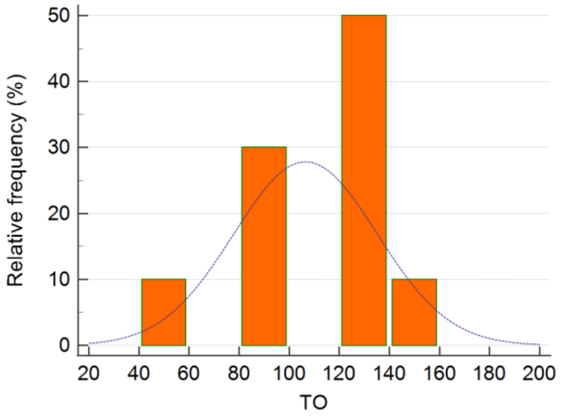

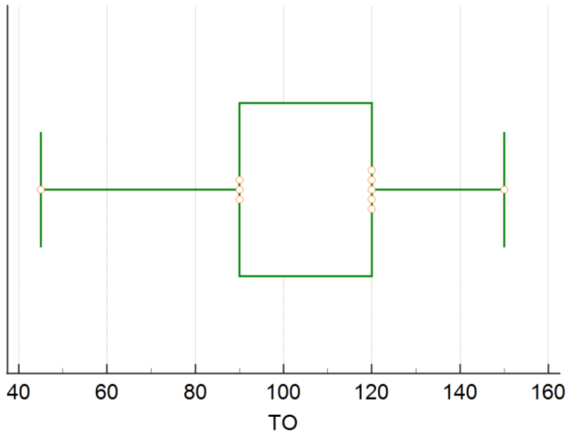

Hair saloons (lower limit of the range)

Sample size	14
Lowest value	10.0000
Highest value	60.0000
Arithmetic mean	34.2857
95% CI for the Arithmetic mean	26.3709 to 42.2005
Median	30.0000
95% CI for the median	25.0000 to 45.0000
Variance	187.9121
Standard deviation	13.7081
Relative standard deviation	0.3998 (39.98%)
Standard error of the mean	3.6636
Coefficient of Skewness	-0.009065 (P=0.9872)
Coefficient of Kurtosis	-0.3560 (P=0.8912)
Shapiro-Wilk test for Normal distribution	W=0.9308 accept Normality (P=0.3133)

Percentiles		95% Confidence interval
2.5		
5	11.0000	
10	14.5000	
25	25.0000	10.7681 to 30.0000
75	45.0000	30.0000 to 57.6957
90	46.5000	
95	57.0000	
97.5		

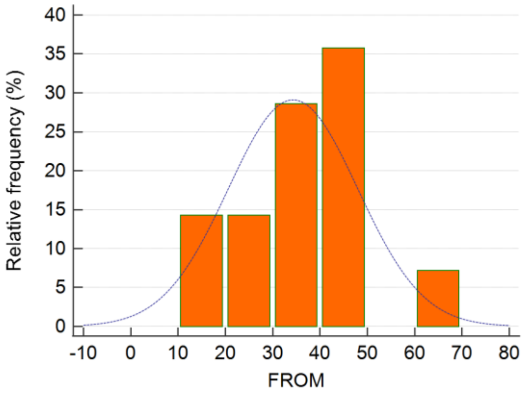

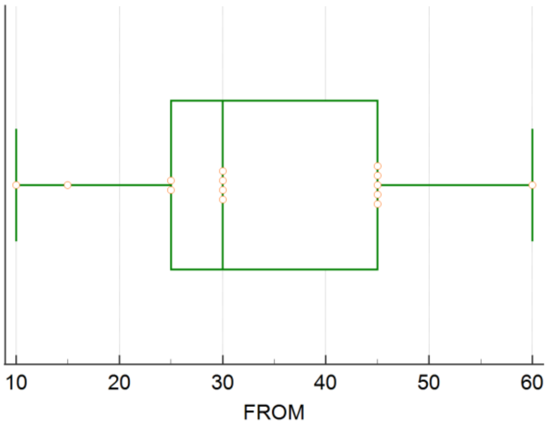

Hair saloons (upper limit of the range)

Sample size	14
Lowest value	60.0000
Highest value	180.0000
Arithmetic mean	100.7143
95% CI for the Arithmetic mean	77.5675 to 123.8611
Median	90.0000
95% CI for the median	60.0000 to 123.1275
Variance	1607.1429
Standard deviation	40.0892
Relative standard deviation	0.3980 (39.80%)
Standard error of the mean	10.7143
Coefficient of Skewness	0.5825 (P=0.3121)
Coefficient of Kurtosis	-0.7370 (P=0.5525)
Shapiro-Wilk test for Normal distribution	W=0.8780 accept Normality (P=0.0544)

Percentiles		95% Confidence interval
2.5		
5	60.0000	
10	60.0000	
25	60.0000	60.0000 to 90.0000
75	120.0000	90.0000 to 175.3914
90	153.0000	
95	174.0000	
97.5		

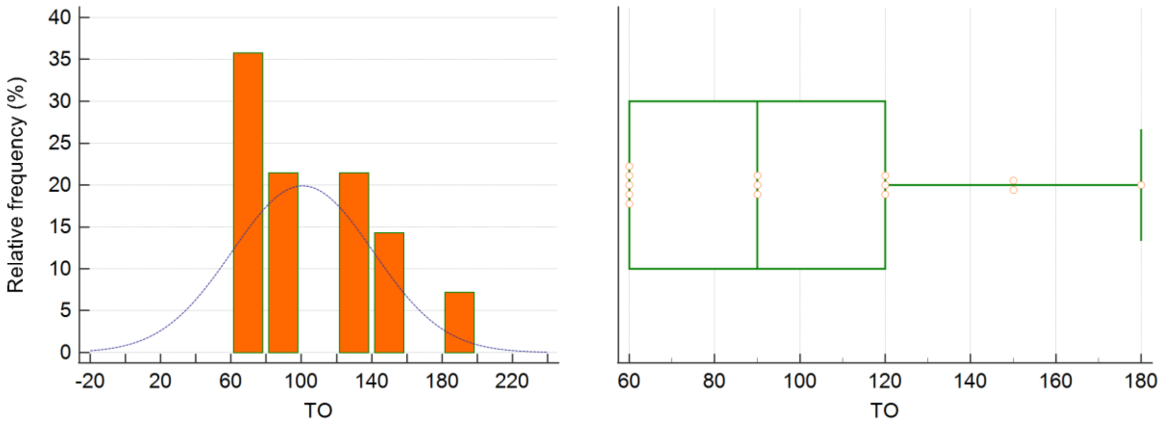

Banks (lower limit of the range)

Sample size	38
Lowest value	10.0000
Highest value	30.0000
Arithmetic mean	16.3158
95% CI for the Arithmetic mean	14.7551 to 17.8765
Median	15.0000
95% CI for the median	15.0000 to 15.0000
Variance	22.5462
Standard deviation	4.7483
Relative standard deviation	0.2910 (29.10%)
Standard error of the mean	0.7703
Coefficient of Skewness	0.8316 (P=0.0341)
Coefficient of Kurtosis	0.7926 (P=0.2517)
Shapiro-Wilk test for Normal distribution	W=0.8577 reject Normality (P=0.0002)

Percentiles		95% Confidence interval
2.5	10.0000	
5	10.0000	
10	10.0000	10.0000 to 15.0000
25	15.0000	10.0000 to 15.0000
75	20.0000	15.0000 to 20.0000
90	23.5000	20.0000 to 29.2540
95	25.0000	
97.5	27.7500	

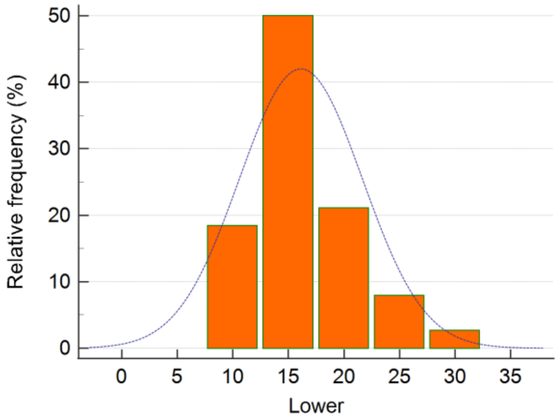

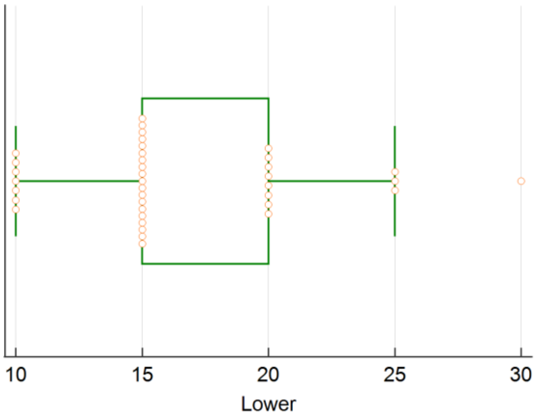

Banks (upper limit of the range)

Sample size	14
Lowest value	45.0000
Highest value	90.0000
Arithmetic mean	49.2857
95% CI for the Arithmetic mean	42.1370 to 56.4345
Median	45.0000
95% CI for the median	45.0000 to 45.0000
Variance	153.2967
Standard deviation	12.3813
Relative standard deviation	0.2512 (25.12%)
Standard error of the mean	3.3090
Coefficient of Skewness	3.2046 (P<0.0001)
Coefficient of Kurtosis	10.5583 (P=0.0002)
Shapiro-Wilk test for Normal distribution	W=0.4106 reject Normality (P<0.0001)

Percentiles		95% Confidence interval
2.5		
5	45.0000	
10	45.0000	
25	45.0000	45.0000 to 45.0000
75	45.0000	45.0000 to 85.3914
90	63.0000	
95	84.0000	
97.5		

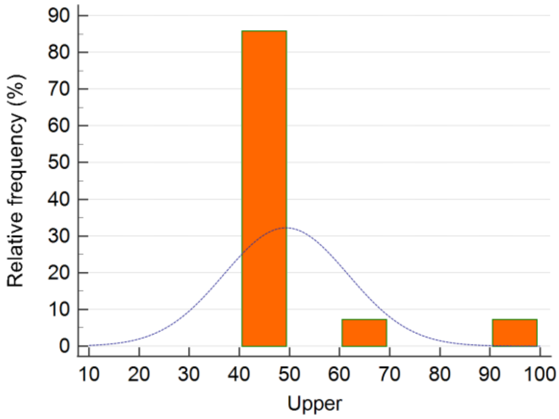

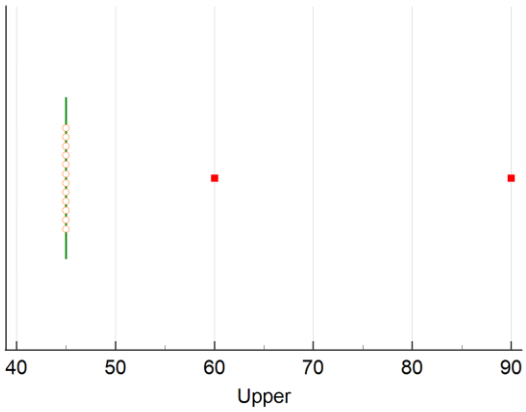

Post offices (lower limit of the range)

Sample size	57
Lowest value	10.0000
Highest value	25.0000
Arithmetic mean	17.7193
95% CI for the Arithmetic mean	16.7788 to 18.6597
Median	15.0000
95% CI for the median	15.0000 to 20.0000
Variance	12.5627
Standard deviation	3.5444
Relative standard deviation	0.2000 (20.00%)
Standard error of the mean	0.4695
Coefficient of Skewness	0.3074 (P=0.3161)
Coefficient of Kurtosis	-0.2394 (P=0.8115)
Shapiro-Wilk test for Normal distribution	W=0.8217 reject Normality (P<0.0001)

Percentiles		95% Confidence interval
2.5	10.0000	
5	15.0000	
10	15.0000	11.0767 to 15.0000
25	15.0000	15.0000 to 15.0000
75	20.0000	20.0000 to 20.0000
90	20.0000	20.0000 to 25.0000
95	25.0000	
97.5	25.0000	

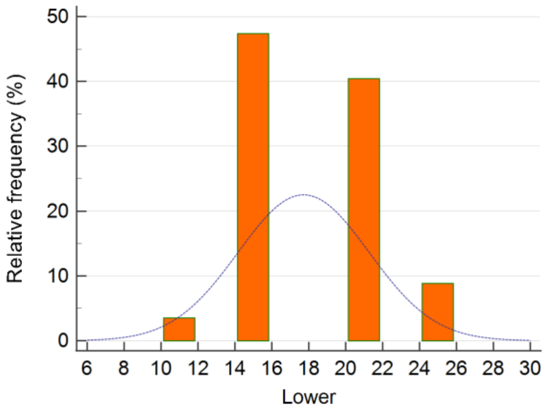

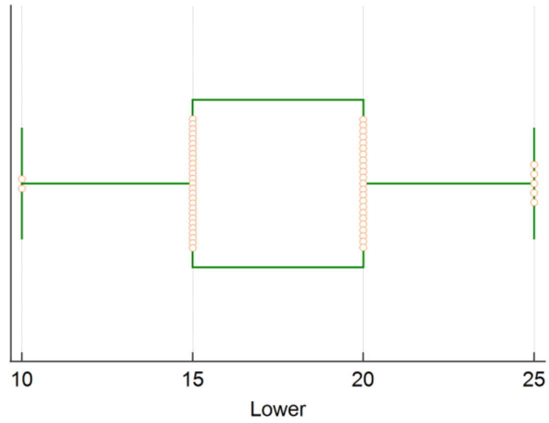

Post offices (upper limit of the range)

Sample size	11
Lowest value	45.0000
Highest value	60.0000
Arithmetic mean	46.3636
95% CI for the Arithmetic mean	43.3253 to 49.4020
Median	45.0000
95% CI for the median	45.0000 to 45.0000
Variance	20.4545
Standard deviation	4.5227
Relative standard deviation	0.09755 (9.75%)
Standard error of the mean	1.3636
Coefficient of Skewness	3.3166 (P<0.0001)
Coefficient of Kurtosis	11.0000 (P=0.0002)
Shapiro-Wilk test for Normal distribution	W=0.3450 reject Normality (P<0.0001)

Percentiles		95% Confidence interval
2.5		
5	45.0000	
10	45.0000	
25	45.0000	
75	45.0000	
90	51.0000	
95	59.2500	
97.5		

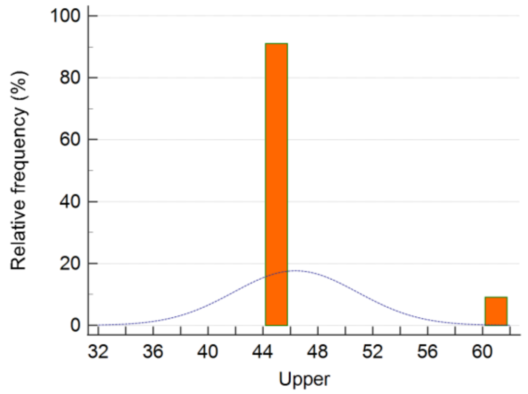

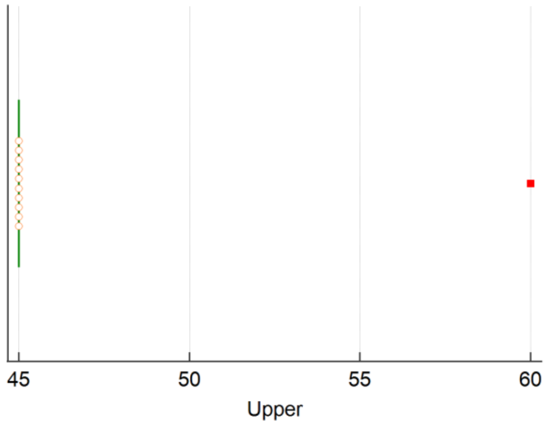

Pharmacies

Sample size	35
Lowest value	10.0000
Highest value	20.0000
Arithmetic mean	14.2857
95% CI for the Arithmetic mean	13.4378 to 15.1336
Median	15.0000
95% CI for the median	15.0000 to 15.0000
Variance	6.0924
Standard deviation	2.4683
Relative standard deviation	0.1728 (17.28%)
Standard error of the mean	0.4172
Coefficient of Skewness	-0.3493 (P=0.3600)
Coefficient of Kurtosis	0.9712 (P=0.2013)
Shapiro-Wilk test for Normal distribution	W=0.6639 reject Normality (P<0.0001)

Percentiles		95% Confidence interval
2.5	10.0000	
5	10.0000	
10	10.0000	
25	15.0000	10.0000 to 15.0000
75	15.0000	15.0000 to 15.0000
90	15.0000	
95	18.7500	
97.5	20.0000	

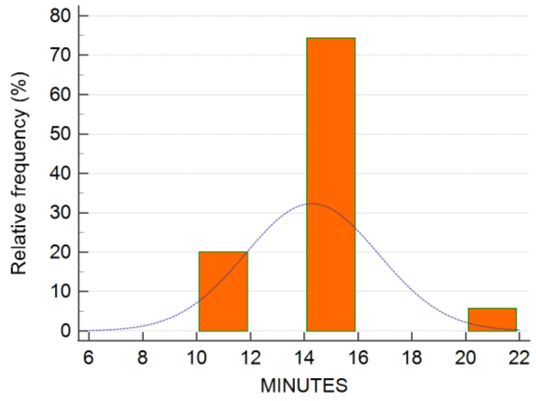

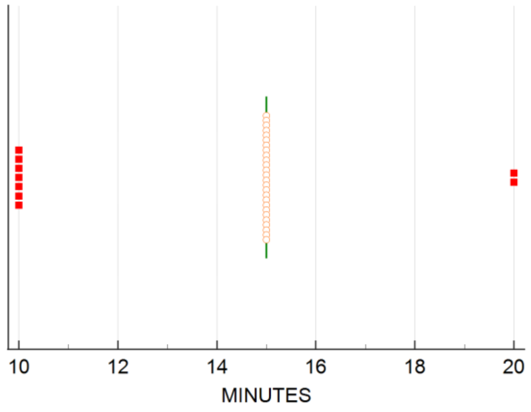

Gas stations

Sample size	20
Lowest value	10.0000
Highest value	15.0000
Arithmetic mean	10.5000
95% CI for the Arithmetic mean	9.7797 to 11.2203
Median	10.0000
95% CI for the median	10.0000 to 10.0000
Variance	2.3684
Standard deviation	1.5390
Relative standard deviation	0.1466 (14.66%)
Standard error of the mean	0.3441
Coefficient of Skewness	2.8879 (P<0.0001)
Coefficient of Kurtosis	7.0370 (P=0.0010)
Shapiro-Wilk test for Normal distribution	W=0.3512 reject Normality (P<0.0001)

Percentiles		95% Confidence interval
2.5	10.0000	
5	10.0000	
10	10.0000	
25	10.0000	10.0000 to 10.0000
75	10.0000	10.0000 to 14.9127
90	12.5000	
95	15.0000	
97.5	15.0000	

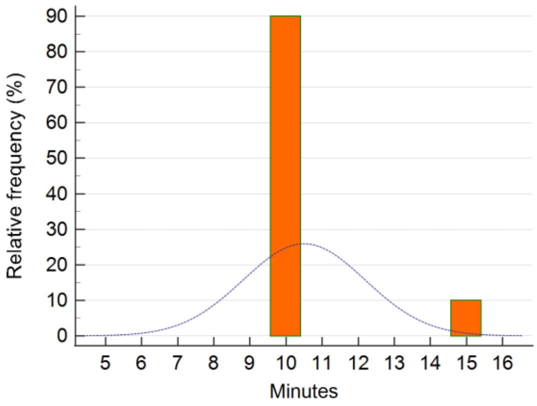

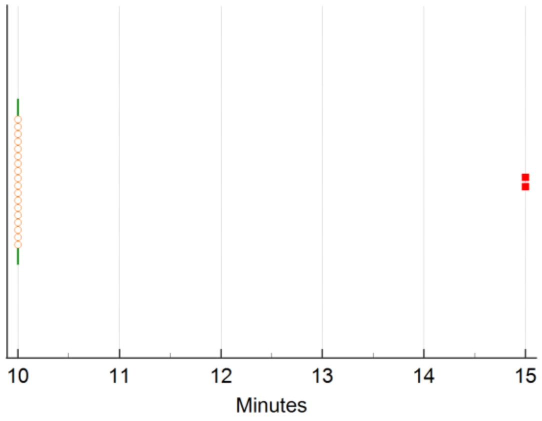

Supplement: Supplementary file 1 [file ijerph-18-04632-s001.zip › ijerph-1174334-supplementary.pdf]
